# Synergistic Sensitization of High-Grade Serous Ovarian Cancer Cells Lacking Caspase-8 Expression to Chemotherapeutics Using Combinations of Small-Molecule BRD4 and CDK9 Inhibitors

**DOI:** 10.3390/cancers16010107

**Published:** 2023-12-24

**Authors:** Khayal Gasimli, Monika Raab, Ranadip Mandal, Andrea Krämer, Samuel Peña-Llopis, Morva Tahmasbi Rad, Sven Becker, Klaus Strebhardt, Mourad Sanhaji

**Affiliations:** 1 Department of Gynecology, University Hospital Frankfurt am Main, 60590 Frankfurt am Main, Germany; khayal.gasimli@kgu.de (K.G.); monika.raab@kgu.de (M.R.); ranadip.mandal@yahoo.com (R.M.); andrea.kraemer@kgu.de (A.K.);; 2 Translational Genomics, Department of Ophthalmology, University Hospital Essen, 45147 Essen, Germany; samuel.pena-llopis@uk-essen.de; 3 German Cancer Consortium (DKTK), 45147 Essen, Germany; 4 German Cancer Research Center (DKFZ), 69120 Heidelberg, Germany; 5 German Cancer Consortium (DKTK), 60590 Frankfurt am Main, Germany

**Keywords:** HGSOC, Caspase-8, CDK9, BRD4, Carboplatin, Paclitaxel, chemotherapeutics, combination therapy

## Abstract

**Simple Summary:**

Despite the recent advancement in the treatment of ovarian cancer, it continues to be a largely incurable disease, plagued by de novo and acquired resistance to standard chemotherapeutics and novel second-line therapeutics. Therefore, identifying novel biomarkers and resistance mechanisms is critical to overcoming resistance, developing newer treatment strategies, and improving patient survival. In this study, we have demonstrated that low Caspase-8 expression correlates with poor prognosis in ovarian cancer patients. Moreover, the lack of Caspase-8 alters transcriptional regulation in ovarian cancer cells. In Caspase-8 knockout cells, increased BRD4 expression, increased CDK9 activity, and resistance to Carboplatin and Paclitaxel were observed. We have also shown that combining BRD4 inhibitors with Carboplatin, Paclitaxel, and CDK9 inhibitors synergistically sensitized these cells to undergo cell death.

**Abstract:**

Ovarian cancer is one of the most lethal gynecological cancers worldwide, with approximately 70% of cases diagnosed in advanced stages. This late diagnosis results from the absence of early warning symptoms and is associated with an unfavorable prognosis. A standard treatment entails a combination of primary chemotherapy with platinum and taxane agents. Tumor recurrence following first-line chemotherapy with Carboplatin and Paclitaxel is detected in 80% of advanced ovarian cancer patients, with disease relapse occurring within 2 years of initial treatment. Platinum-resistant ovarian cancer is one of the biggest challenges in treating patients. Second-line treatments involve PARP or VEGF inhibitors. Identifying novel biomarkers and resistance mechanisms is critical to overcoming resistance, developing newer treatment strategies, and improving patient survival. In this study, we have determined that low Caspase-8 expression in ovarian cancer patients leads to poor prognosis. High-Grade Serous Ovarian Cancer (HGSOC) cells lacking Caspase-8 expression showed an altered composition of the RNA Polymerase II-containing transcriptional elongation complex leading to increased transcriptional activity. Caspase-8 knockout cells display increased BRD4 expression and CDK9 activity and reduced sensitivities to Carboplatin and Paclitaxel. Based on our work, we are proposing three potential therapeutic approaches to treat advanced ovarian cancer patients who exhibit low Caspase-8 expression and resistance to Carboplatin and/or Paclitaxel—combinations of (1) Carboplatin with small-molecule BRD4 inhibitors; (2) Paclitaxel with small-molecule BRD4 inhibitors, and (3) small-molecule BRD4 and CDK9 inhibitors. In addition, we are also proposing two predictive markers of chemoresistance—BRD4 and pCDK9.

## 1. Introduction

Ovarian cancer is one of the most lethal gynecological cancers in the world. Due to the lack of early warning symptoms, over 70% of ovarian cancer cases are diagnosed at advanced stages and are largely incurable, resulting in a poor prognosis [1,2]. The 5-year survival rate presently stands at 47.4%. The most common type (95%) of ovarian cancer is of epithelial origin, which is histologically further classified into serous (77%), endometroid (10%), mucinous (3%), and clear-cell (10%) sub-types. The serous sub-types are further classified into HGSOC (<90%), in which the *TP53* gene is most frequently mutated (96%) [3], and Low-Grade Serous Ovarian Carcinomas (LGSOC) (~5%). LGSOC has a better prognosis and is usually diagnosed at younger ages. In contrast, HGSOC has an inferior prognosis, detected at later stages in 85% of women, and a 10-year mortality rate of 70% [4,5].

For over two decades, the established therapeutic approach for ovarian cancers has encompassed extensive cytoreductive surgery, succeeded by a regimen combining platinum-taxane-based primary chemotherapy. Following these treatment protocols, most patients achieve a complete clinical remission. However, 70% of patients diagnosed at FIGO stages III or IV suffer from subsequent recurrence and chemoresistance within the first 5 years [1,5,6]. Advanced patients, where cytoreductive surgery is impossible, are first treated with neoadjuvant chemotherapy, followed by interval cytoreduction [3,7]. Regrettably, late-stage ovarian cancer cases remain incurable. Nevertheless, the ongoing research is dedicated to the development and clinical evaluation of novel pharmaceutical agents targeting pivotal molecular pathways associated with cancer cell proliferation, tumor growth, immune evasion, and apoptosis. While anti-angiogenic factors and PARP inhibitors have already been approved by the FDA, folate receptor inhibitors, and immunotherapeutic strategies are still under clinical investigation [3,5]. These new approaches stabilize the disease or delay its recurrence but fail to deliver a definitive cure.

The second-line therapy of ovarian cancer patients who develop a recurrence depends on their sensitivity towards platinum derivatives. Highly or partially sensitive tumors are usually treated with Carboplatin or Cisplatin in combination with Paclitaxel, Pegylated Liposomal Doxorubicin (PLD), or Gemcitabine (with or without Bevacizumab). When partially sensitive patients cannot be treated with platinum, PLD in combination with Trabectedin is recommended [3]. Platinum-resistant patients have a poor prognosis and do not benefit from monotherapy or combination PLD, Gemcitabine, or Paclitaxel therapies. In some patients, combining chemotherapy with Bevacizumab, a recombinant humanized monoclonal antibody against VGEF, can prolong Progression-Free Survival (PFS) [3,5,8,9]. Thus, the identification of predictive markers and resistance mechanisms is of paramount importance for the development of novel therapeutic strategies against ovarian cancer.

Transcriptional elongation is a complex process in gene expression, tightly regulated by RNA Polymerase II (RNAPII) and various factors (Supplementary Figure S1a) [10]. In the context of cancer, the dysregulation of transcriptional elongation holds profound implications, with BRD4 (Bromodomain-containing protein 4) emerging as a pivotal contributor in this context (Supplementary Figure S1b) [11]. Cancer often involves aberrant gene expression, and the defective transcriptional elongation can be a contributing factor. BRD4, a protein featuring bromodomains that selectively recognize acetylated histones, is frequently found to be overexpressed in cancer [12]. Its upregulation can lead to the increased recruitment of RNAPII to gene promoters and enhancers, resulting in the elevated transcription of oncogenes. Moreover, BRD4’s interaction with the positive transcription elongation factor b (P-TEFb), which consists of Cyclin-dependent kinase 9 (CDK9), in tandem with one of the corresponding Cyclin T1, T2, or K, helps RNAPII escape the promoter-proximal pausing, allowing for the continuous transcription of genes critical to cancer progression [13]. This process can lead to uncontrolled cell growth and proliferation. Targeting BRD4 in cancer therapy has gained attention due to its role in transcriptional regulation. Inhibitors that disrupt the BRD4-P-TEFb interaction have shown promise in halting cancer cell growth and reducing tumor progression. In summary, the interplay between transcriptional elongation, RNAPII, BRD4, and cancer is a dynamic and intricate process. Understanding these interactions may provide insights into cancer mechanisms and offer potential therapeutic strategies for combating this devastating disease.

CDK9 and RNAP II are critical components of the transcription elongation machinery, including several essential proteins. Key components are RNA Polymerase (e.g., RNAPol II in eukaryotes), which catalyzes RNA synthesis, and the C-Terminal Domain (CTD) of its largest subunit. Factors like P-TEFb (Positive Transcription Elongation Factor b) help overcome promoter-proximal pausing, while NELF (Negative Elongation Factor) regulates elongation rate [10]. Additionally, transcription factors like TFIIS and TFIIF support the efficient transcription. Chromatin-modifying enzymes, such as histone acetyltransferases (HATs) and histone methyltransferases (HMTs), are crucial for chromatin remodeling during elongation [14]. These proteins form a complex network that orchestrates transcription elongation, ensuring proper gene expression and RNA production. Negative and positive regulators like NELF-A (Negative Elongation Factor A) and Larp-7 (La-Related Protein 7) influence elongation dynamics. NELF-A, a part of the NELF complex, induces the pausing of RNA Polymerase II near the transcription start site, temporarily halting transcription Larp-7 collaborates with Hexim1, forming a complex that controls P-TEFb activity, a positive elongation factor. This regulation ensures the precise timing for RNA Polymerase II release from the pause, allowing transcription to continue. Spt5 (Suppressor of Ty5), part of the DSIF complex, can either hinder or stimulate elongation, depending on specific factors and conditions, further modulating the transcription [15]. In our prior work, we substantiated that in cervical cancer patients exhibiting a high Tumor Mutational Burden (TMB), diminished Caspase-8 expression was associated with a dismal prognosis and resistance to conventional chemotherapeutic agents, specifically Carboplatin and Cisplatin. This resistance mechanism was attributed to the heightened activation and activity of CDK9 (phosphorylated CDK9) [pCDK9]. Our investigation unveiled a non-apoptotic role for Caspase-8, demonstrating its function as an inhibitor of CDK9. This inhibition led to the augmented phosphorylation of RNAPII at Ser2, resulting in the altered expression of genes governing chemoresistance and cellular migration. Notably, our research underscored that combining a small-molecule CDK9 inhibitor with Cisplatin elicited a synergistic inhibition of the cervical cancer cell growth under both 2D and 3D culture conditions, particularly in cases lacking Caspase-8 expression [16,17].

In this study, we examined the role of Caspase-8 expression in HGSOC cell lines to identify potential predictive markers, such as BRD4. We tested combinations of small-molecule CDK9 and/or BRD4 inhibitors in conjunction with Carboplatin and Paclitaxel and observed a synergistic inhibition of HGSOC ovarian cancer cell growth.

## 2. Materials and Methods

### 2.1. Cell Culture

The OVCAR-3 were kindly provided from Dr. David Bowtell’s lab. OVCAR-8 cell lines were obtained from the DCTD tumor repository (Frederick, MD, USA) and cultured according to their instructions. Cells were cultivated in RPMI 1640, 10% FCS, and 1% Pen/Strep.

### 2.2. Antibodies, Reagents, siRNAs, and Plasmids

Antibodies and sources: CDK9 (1.1000), pCDK9 (Thr187) (1.1000), RNAPII (1.1000), phospho-RNAPII(Ser2) (1.1000), PARP (1.1000), and NUP98 (1.1000) (Cell Signaling Technology); HEXIM1 (1.1000), NELF-a (1.1000), LARP-7 (1.1000), c-Myc (1.1000), SPT5 (1.1000), and GAPDH (1.5000) (Santa Cruz Biotechnology, Dallas, TX, USA); BRD4 (1.1000) (Abcam, Waltham, MA, USA); Caspase-8 (1.1000) (Enzo Life Sciences, Farmingdale, NY, USA); β-Actin (1.10000), (Sigma-Aldrich, St. Louis, MO, USA); Cyclin T (1.1000) (Bethyl, Montgomery, TX, USA). Reagents and sources: CellTiter-Blue Cell Viability assay and Caspase-Glo 3/7 assays (Promega, Madison, WI, USA); AnnexinV and 7AAD (BD); BAY1251152, BI894999, ABBV744, Paclitaxel and Carboplatin (Selleckchem, Houston, TX, USA); BioCoat Matrigel invasion chamber (Corning, Corning, NY, USA); Migration chamber (Ibidi, Gräfelfing, Germany); RNeasy Plus kit (Qiagen, Venlo, The Netherlands). The following vectors were used: pCas9(BB)-2A-Puro (PX459) V2.0 (62988, Addgene, Watertown, MA, USA); p3xFlag-CMV-7.1 (E7533, Sigma, Ronkonkoma, NY, USA).

### 2.3. Western Blot

Cell protein extracts were generated through cell lysis in RIPA buffer (Sigma) containing protease inhibitors (Complete protease inhibitor cocktail, Roche, Atlanta, GA, USA). Subsequently, 25 μg of protein extracts were subjected to separation via SDS-PAGE and transferred onto PVDF membranes utilizing the TransBlot Turbo Transfer System (BioRad, Benicia, CA, USA). Membrane blocking was conducted using TBST with 2% BSA.

### 2.4. CRISPR/Cas9-Mediated Stable Knock-Out of CASP8 and Caspase-8 Downregulation by siRNA

As previously outlined [17], we achieved a stable knockout of the CASP8 gene in OVCAR-3 and OVCAR-8 cells by targeting two specific regions within exon 1 of Caspase-8. This was accomplished using the (PX459) plasmid, with the designated target sequences as follows: (1) 5′-GCC TGG ACT ACA TTC CGC AAAGG-3′ and (2) 5′-GCT CTT CCG AAT TAA TAG ACTGG-3′. Positive clones were confirmed through sequencing. The knock down of Caspase-8 was achieved using siRNA targeting the following sequence: GAGCUAAAGUUAAAUAGGAUU.

### 2.5. Immunoprecipitation

Cell lysates were incubated with Protein G Sepharose beads (GE Healthcare, Alger, OH, USA) and the specific antibody overnight at 4 °C. Immunoprecipitates were washed 3× with an ice-cold buffer (20 mM Tris-HCl [pH 8.2], 150 mM NaCl, 1% [*v*/*v*] TritonX-100).

### 2.6. Subcellular Fractionation

All procedures were conducted on ice. Cells were washed with PBS, harvested, and suspended in hypotonic buffer (20 mM Tris-HCl, pH 7.4, 10 mM KCl, 2 mM MgCl_2_, 1 mM EGTA, 0.5 mM DTT, 0.5 mM PMSF, and Roche complete protease inhibitors; 300 μL per 100-mm tissue culture dish). After a 5-min incubation, NP-40 (Nonidet P-40) was added to achieve a final concentration of 0.1%. Following a 3 min incubation, cytoplasmic and nuclear fractions were separated by centrifugation at 800× *g* for 8 min. To ensure the removal of nuclear remnants, the cytoplasmic fractions were further centrifuged at 1500× *g* for 5 min, and the resulting supernatants were collected as the final cytoplasmic fractions. Nuclei were purified by a 10-min incubation in isotonic lysis buffer (20 mM Tris-HCl, pH 7.4, 150 mM KCl, 2 mM MgCl_2_, 1 mM EGTA, 0.3% NP-40, 0.5 mM DTT, and 0.5 mM PMSF) with Roche complete protease inhibitors, followed by centrifugation at 700× *g* for 7 min.

### 2.7. Analysis of Apoptosis

The Vybrant Apoptosis Assay Kit (Alexa Fluor 488 annexin V/propidium iodide staining, Invitrogen, Carlsbad, CA, USA) was employed, and flow cytometry analysis was conducted using a FACSCalibur (BD Biosciences, San Jose, CA, USA). Caspase-3/7 activity was assessed using the Caspase-Glo 8 or Caspase-Glo 3/7 Assay (Promega). Each well received 20 microliters of substrate and 1 µg of protein, followed by 30 min of shaking at room temperature in the dark, with luminescence detected using Victor X4 (Perkin Elmer, Waltham, MA, USA).

### 2.8. Phenotypic Analysis

The 2D cell migration: 0.1 × 10^6^ cells were seeded in 70 µL medium on each side of an ibidi migration chamber and left to settle overnight. After removing the chambers and flooding the wells with medium, cell migration between the two populations from each chamber was monitored using time-lapse brightfield microscopy. The reductions in the area between the two populations at each time point were then quantified.

The 3D cell invasion: 5 × 10^4^ cells were seeded in Matrigel-coated invasion chambers in a serum-starved medium. The chambers were placed in 12-well plates, also filled with serum-starved medium. Following overnight culture, only the medium in the wells was replaced with FCS-containing medium to allow the cells from the chamber to migrate toward this medium through the Matrigel for 24 h. The invaded cells were fixed and permeabilized, and their nuclei were stained with DAPI to be observed with fluorescence microscopy.

### 2.9. Cell Proliferation Assay

Cell viability and proliferation assays were conducted using the CellTiter-Blue Cell Viability Assay (Promega). Cells were seeded in 96-well plates, and the fluorescence was measured using a Victor Victor X4 (Perkin Elmer).

### 2.10. Statistical Analysis

For the analysis of The Cancer Genome Atlas (TCGA), data encompassing RNA-Seq, Reverse Phase Protein Array (RPPA), and clinical information associated with ovarian serous adenocarcinoma (OV) patients were sourced from the TCGA database (https://portal.gdc.cancer.gov/ (accessed on 15 August 2018)). The data underwent scrutiny following methods outlined elsewhere [17,18]. The average of the z scores for RNA-Seq and/or RPPA were stratified according to quartiles, where the 2nd, 3rd and 4th quartiles were combined into intermediate (2nd and 3rd quartiles) and high expression (4th quartile). Kaplan–Meier survival curves and log-rank tests, and Cox regressions were performed using IBM SPSS Statistics v.25 (IBM, Ehningen, Germany). Comparisons of low vs. high CASP8 expression (1st vs. 4th quartile) with clinical variables were calculated using Fisher’s Exact tests.

To determine the Combination Index (CI), the cell-proliferation assay results were subjected to calculation using the Chou–Talalay method [19]. The Compusyn v.1 software was employed for this computation, following the guidelines provided by the developers [20].

## 3. Results

### 3.1. Low Expression of Caspase-8 Correlates with Poor Prognosis

**Figure 1:**
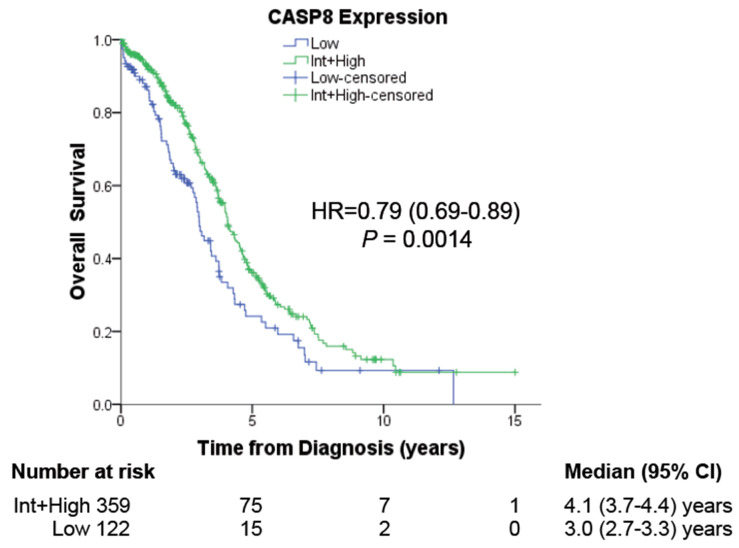
The effect of *CASP8* expression on patient prognosis. RNA-Seq and Reverse Phase Protein Array (RPPA) expression data for *CASP8* of 481 ovarian cancer patients were obtained from the TCGA database (OV-TCGA) and used to determine the correlation between high or low *CASP8* expression with their Overall Survival (OS).

To determine the significance of *CASP8* expression in the prognosis of ovarian cancer patients, we analyzed the association of *CASP8* expression with clinical variables from TCGA for ovarian serous adenocarcinoma (OV-TCGA). Our examination encompassed 481 patients with available RNA-seq and Reverse Phase Protein Array (RPPA) data for CASP8. The results unveiled a significant association between low CASP8 expression and diminished Overall Survival (OS), as depicted in Figure 1. In our earlier observations in cervical cancer patients with higher Tumor Mutational Burden (TMB), the expression of CASP8 was also of prognostic relevance [17]. Intriguingly, in ovarian cancer patients, low CASP8 expression was correlated with advanced clinical stages, although no corresponding correlation with higher tumor grades was observed (Table 1). In summary, this data establishes the prognostic significance of CASP8 expression for ovarian cancer patients.

### 3.2. Alteration of Cellular Behaviors Due to the Knock-Out of Caspase-8

**Figure 2:**
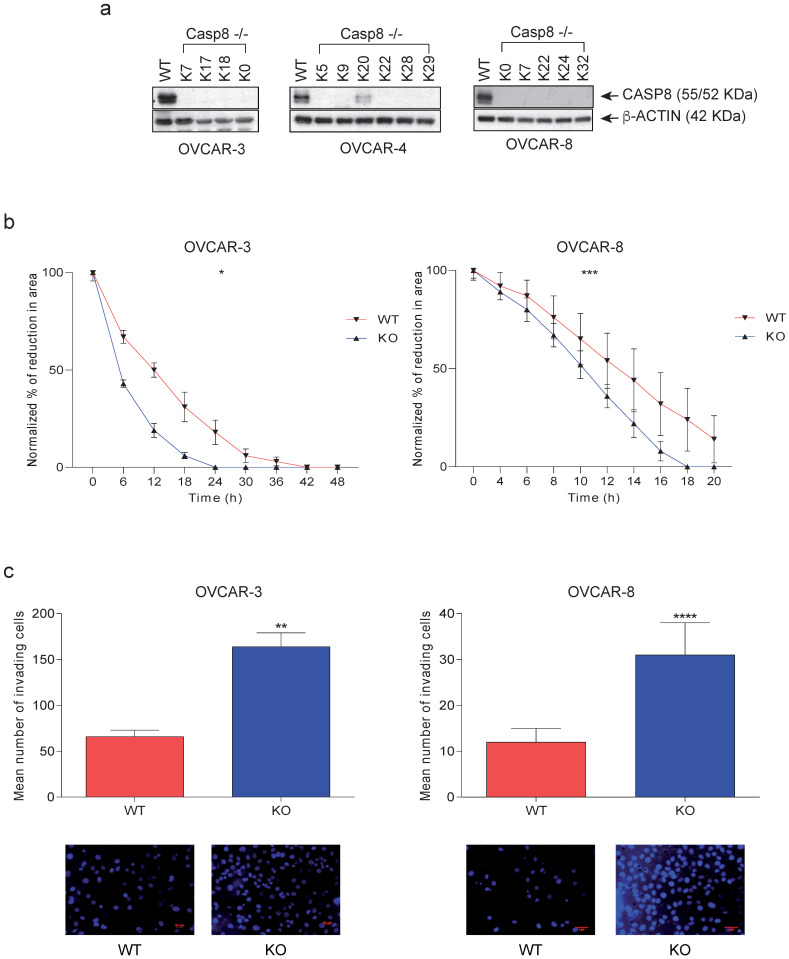
Effects of Caspase-8 knock-out on the behavior of ovarian cancer cell lines. (**a**) HGSOC cell lines OVCAR-3, and OVCAR-8 CASP8^−/−^ knock-out clones were generated using the CRISPR/Cas9 genome editing system. Individual clones [K7, 17, 18, and their mix (KO)] for OVCAR-3 and [K7, 22, 24, 32, and their mix (KO)] for OVCAR-8 were lysed and subjected to a Western blot analysis using Caspase-8 and β-Actin antibodies. (**b**) The 2D migration of OVCAR-3 and OVCAR-8 wt and KO cells was determined using ibidi migration chambers over 48 h and 20 h, respectively. The reductions in the areas between the two cell populations at each time point, representing the migration of the cells, were measured, normalized to the area at 0 h, and represented graphically [mean ± SD; *n* = 3 for each time point; *p*-value (paired *t-*test, two-tailed); * *p* ≤ 0.05; *** *p* ≤ 0.001]. (**c**) The 3D invasion of OVCAR-3 and OVCAR-8 wt and KO cells was determined using Matrigel-coated invasion chambers over 24 h. The nuclei of the invaded cells were stained with DAPI (bottom panel), and their quantification was represented graphically. [mean ± SD; *n* = 3 for each time point; *p*-value (paired *t-*test, two-tailed); ** *p* ≤ 0.01; **** p ≤ 0.0001]. The original western blots of Figure 2a are shown in Figure S7.

**Figure 3:**
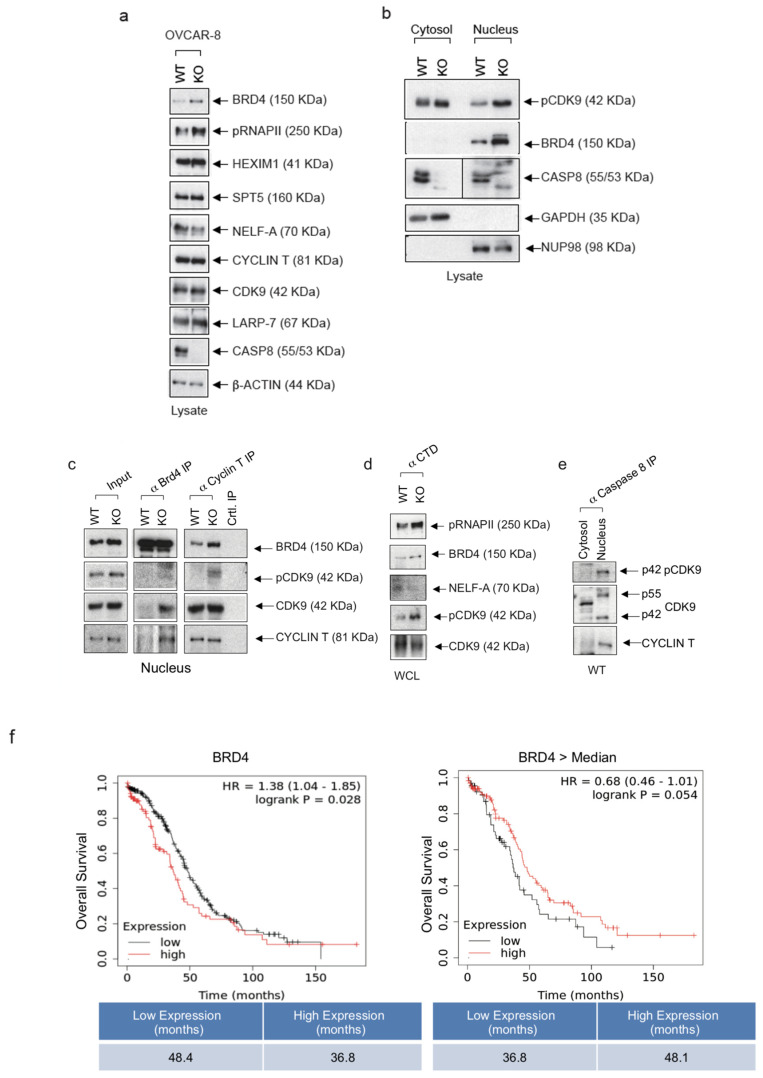
Knock-out of Caspase-8 expression enhances CDK9 phosphorylation and BRD4 expression in ovarian cancer cells. (**a**) OVCAR-8 wt and KO cells were immunoblotted and checked the levels of BRD4, phospho-carboxyterminal domain of RNAPII/pSer2 (pCTD), HEXIM, SPT5, NELF-A, Cyclin T, CDK9, Caspase-8, LARP7, Caspase-8, and GAPDH. (**b**) OVCAR-8 wt and KO cells were fractionated into their cytosol and nuclear fractions, immunoblotted, and checked for levels of pCDK9, BRD4, Caspase-8, GAPDH (cytosolic marker), and NUP98 (nuclear marker). (**c**) Lysates OVCAR-8 wt and KO cells were subjected to anti-BRD4 co-IP or anti-Cyclin T co-IP, and blotted for BRD4, pCDK9, CDK9, and Cyclin T. (*n* = 3). (**d**) Lysates OVCAR-8 wt and KO cells were subjected to anti-CTD co-IP and blotted for pCTD, BRD4, NELF-A, pCDK9, and CDK9. (*n* = 3). (**e**) Lysates OVCAR-8 wt cells separated into cytosolic and nuclear fraction were subjected were subjected to anti-Caspase-8 co-IP and blotted for pCDK9, CDK9, and Cyclin T. (*n* = 3). (**f**) Analysis of the mRNA-Seq data of ovarian cancer patients (all grades) from the kmplot database (www.kmplot.com (accessed on 15 November 2020)) to check the effect of BRD4 expression on the prognosis of these patients. The original western blots are shown in Figure S7.

For an analysis of the biological role of Caspase-8 in HGSOC, we first knocked out the expression of Caspase-8 in three different HGSOC cell lines, OVCAR-3, and OVCAR-8 [21] using the CRISPR/Cas9 system. To avoid clonal variations, we mixed individual *CASP8^−/−^* KO clones, clones K7, 17, and 18 for OVCAR-3 and clones K7, 22, 24, and 32 for OVCAR-8, to form a mixed knock-out population (henceforth KO) (Figure 2a). Our investigation centered on elucidating the relationship between the migratory and invasive behaviors of ovarian cancer cells, considering the presence or absence of Caspase-8 expression. To explore these questions, we employed knock-out (KO) cells alongside their Caspase-8 expressing wild-type (wt) counterparts. The knock-out of *CASP8* in OVCAR-3 and OVCAR-8 led to significantly enhanced 2D cell migration (Figure 2b) and 3D cell invasion (Figure 2c) compared to their respective wt counterparts. These findings shed light on previously unexplored biological roles of Caspase-8 in HGSOC cell lines, demonstrating that the absence of Caspase-8 stimulates the migration and invasiveness of these cells.

### 3.3. Knock-Out of Caspase-8 Expression Enhances the Phosphorylation of CDK9 at Thr187 and the Expression of BRD4 in Ovarian Cancer Cells

Considering the role of Caspase-8 as an inhibitor of transcriptional elongation in cancer cells we explored previously [13], we initiated a molecular investigation in ovarian cancer cells. This analysis focused on the functionally significant components of the multimeric RNAPII complex, including BRD4, HEXIM1, Spt5, NELF-A, Cyclin T, CDK9, LARP-7, and Caspase-8, which collectively serve as a regulatory hub in gene expression in cancer cells. In OVCAR-8 KO cells, we observed an elevated protein expression of BRD4 (Figure 3a), which recruits P-TEFb to the promotor-proximal gene region (Supplementary Figure S1b). Additionally, compared to wt cells, there was an increased phosphorylation of RNAPII-CTD at Ser2 (pCTD), which represents a significant phosphorylation site for CDK9 within RNAPII-CTD (Figure 3a). Additionally, the subcellular fractionation of lysates obtained from OVCAR-8 KO cells revealed a substantial elevation in the phosphorylation of CDK9 at Thr187 (pThr187) and a pronounced enrichment of BRD4 within the nucleus, as opposed to the nuclei of wt cells (Figure 3b).

Furthermore, our analysis was expanded to include OVCAR-3 cells, providing additional support for the observed elevation in BRD4 levels within the nuclei of OVCAR3 knockout cells compared to their wild-type counterparts. However, it should be noted that the proposed control of expression by Caspase-8 is likely through transcriptional regulation, although this mechanism is not explicitly demonstrated. (Supplementary Figure S2a).

Next, we performed co-immunoprecipitations (co-IP) using antibodies for BRD4, Cyclin T or the phosphorylated carboxyterminal end of RNAPII-CTD that includes phosphorylated Ser2 (Figure 3c,d). A major function of BRD4 is the recruitment P-TEFb (CDK9/Cyclin T) to the promoter-proximal gene region through its binding to acetylated chromatin (Supplementary Figure S1b). Notably, BRD4 interacts with P-TRFb via its P-TEFb interaction domain (PID), thereby stimulating its kinase activity and stimulating its phosphorylation of the carboxy-terminal domain (CTD) of RNAP II [22]. The co-IP, using BRD4 antibodies, supported elevated levels of BRD4 in lysates of OVCAR-8 KO cells and clearly more CDK9 with a noticeable increase in CDK9 and Cyclin T within the precipitate (Figure 3c). This observation suggests a pronounced recruitment of the active PTEFb complex to the chromatin. Co-IP with Cyclin T antibodies further confirmed the CDK9 activation by showing increased levels of autophosphorylated CDK9 (pCDK9). Additionally, our model of augmented P-TEFb activity in the regulation of PolII transcription was substantiated through the use of pCTD antibodies, which revealed the intensified activation of CDK9 (pCDK9) and increased phosphorylation of CTD (pCTD) as an endogenous substrate of CDK9 (Figure 3d). These findings were further validated in OVCAR-3 KO cells. Co-IP experiments employing BRD4- or Cyclin T-specific antibodies provided additional support for the robust activation of CDK9, as evidenced by elevated levels of pCDK9 (Supplementary Figure S2b).

Since RNAPII is of paramount importance for transcriptional elongation, we studied the transcription elongation machinery which includes several essential proteins like SPT5, LARP7, HEXIM1, Cyclin T, and BRD4 (Figure 3a–d). The 7SK snRNA molecule plays a crucial role in gene expression regulation by forming a complex with P-TEFb (Supplementary Figure S1a). When 7SK snRNA binds to P-TEFb, it keeps P-TEFb in an inactive state, preventing it from initiating the transcription elongation prematurely (Supplementary Figure S1a). This binding and sequestration of P-TEFb by 7SK snRNA help to maintain a balance in the gene expression, ensuring that transcription is initiated at the right time and in the appropriate context, thereby influencing the overall gene regulation process. While the immunoprecipitation of BRD4 from OVCAR-8 KO cells revealed elevated levels of Cyclin T, suggesting that BRD4 was to be part of the active P-TEFb complex (Figure 3c), the IP targeting RNAPII from wt OVCAR-8 cells using CTD antibodies revealed elevated levels of NELF-A (Figure 3d). This is a component of the NELF complex and induces the pausing of RNAPII near the transcription start site, temporarily halting transcription as compared to OVCAR-8 KO cells. These observations support the hypothesis of Caspase-8 being an inhibitor of the transcriptional elongation complex with the ability to shift it to its inactive form. The IP targeting Caspase-8 using lysates of OVCAR-8 cells provided evidence for an association of Caspase-8 and both isoforms of CDK9 (p55, p42) with its regulatory subunit Cyclin T (Figure 3e).

Considering the emerging view of BRD4 as a general regulator of transcriptional regulation and its prominent role in cancer development, we focused on subsequent investigations on the molecular role of BRD4 in ovarian cancer cells. The analysis of the mRNA-Seq data of ovarian cancer patients (all grades) from the kmplot database (www.kmplot.com (accessed on 15 November 2020)) revealed that the overexpression of BRD4 in ovarian cancer patients led to significantly reduced OS compared to low BRD4 expressing patients (36.8 vs. 48.4 months) (Figure 3f, left panel). We next used the same patient data to demonstrate the correlation between Caspase-8 and BRD4 expression but selected only those with higher than median BRD4 expression. Within this subset, our analysis unveiled that in cases with elevated BRD4 expression, the lower Caspase-8 expression was associated with a notable reduction in OS compared to cases with a high Caspase-8 expression (36.8 vs. 48.1 months) (Figure 3f, right panel). In summary, these findings lead us to conclude that the BRD4 expression is elevated in HGSOC cell lines lacking the Caspase-8 expression, and the overexpression of BRD4 significantly shortens OS in ovarian cancer patients, mainly when the Caspase-8 expression is low.

### 3.4. Loss of Caspase-8 Expression Imparts Resistance towards Chemotherapeutics and Small-Molecule CDK9 and BRD4 Inhibitors

**Figure 4:**
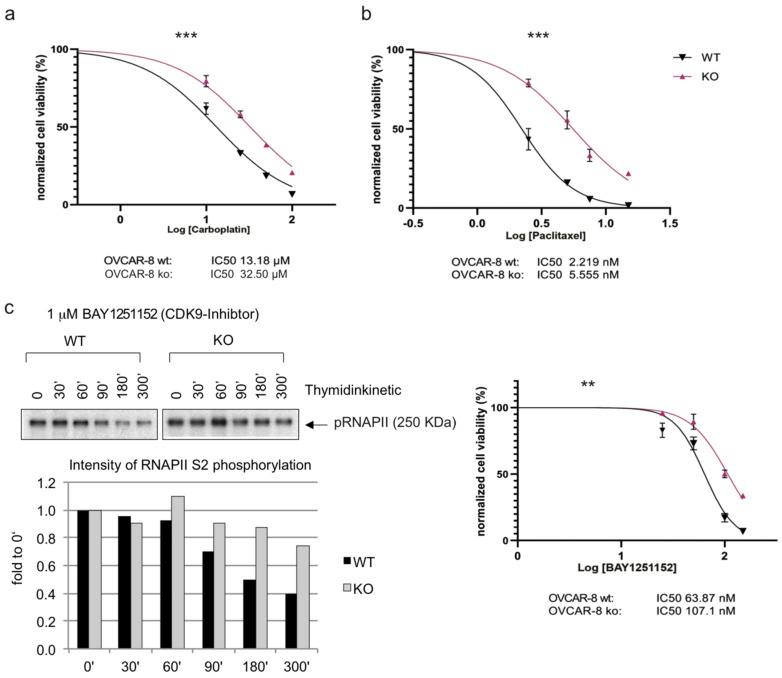

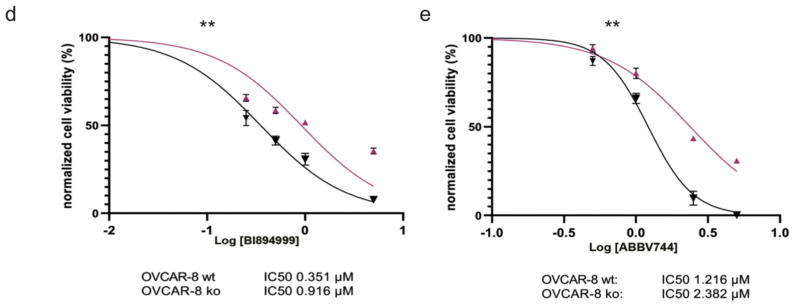
Effects of Caspase-8 knock-out on the response of ovarian cancer cells to Carboplatin, Paclitaxel, and small-molecule inhibitors of CDK9 and BRD4. OVCAR-8 wt and KO cells were treated with increasing concentrations of (**a**) Carboplatin, (**b**) Paclitaxel, and the small-molecule inhibitors (**c**) (**right**) CDK9 inhibitor BAY1251152, (**d**) BRD4 inhibitor BI894999 or (**e**) BRD4 inhibitor ABBV744. The corresponding proliferation was measured using a CellTiter-Blue Cell Viability assay over a period of 96 h, with measurements taken every 24 h. The proliferation rates of the treated cells, an indication of cell viability, were normalized with their respective DMSO-treated counterparts (vehicle control) and subsequently used to calculate the IC_50_ values of each cell type. The plots represent the IC_50_ values at the 96 h time point. [*n* = 3]. Displayed are the results of one representative experiment as mean ± SD; (two-way Anova); ** *p* ≤ 0.01; *** *p* ≤ 0.001]. (**c**) (**left**) OVCAR-8 wt and KO cells were treated with 1 µM BAY1251152 (CDK9 inhibitor) and incubated for the indicated time points up to 300 min. The cells were lysed, and the phosphorylation status of the RNAPII/pSer2 (pCTD) was assessed using Western blot. The pCTD signal was quantified and displayed as a bar graph. The original western blots of Figure 4c are shown in Figure S7.

The treatment with Carboplatin or Cisplatin in combination with Paclitaxel is part of the standard chemotherapy employed to treat ovarian cancer [3]. Therefore, our next objective was to establish the sensitivity of HGSOC cell lines in the presence or absence of the Caspase-8 expression to standard therapy. To investigate this, we subjected OVCAR-8 wt and CASP8^−/−^ KO cells to escalating concentrations of Carboplatin (Figure 4a, Supplementary Figure S3a) or Paclitaxel (Figure 4b, Supplementary Figure S3b) and assessed their proliferation over 96 h. Our observations revealed that OVCAR-8 KO cells exhibited a significant reduction in sensitivity to both Carboplatin and Paclitaxel in comparison to their wt counterparts (IC_50_ wt vs. KO: 13.18 vs. 32.50 µM for Carboplatin and 2.22 vs. 5.56 nM for Paclitaxel) (Figure 4a,b, Supplementary Figure S3a,b).

Due to the increased activity of CDK9 in OVCAR-8 KO and OVCAR-3 KO cells (Figure 3, Supplementary Figure S2), we also determined the sensitivity of these cells to the small-molecule CDK9 inhibitor BAY1251152 (BAY) [23]. Initially, we examined the impact of BAY on CDK9 activity and the phosphorylation of Ser2 within its endogenous substrate RNAPII. The titration experiment showed the inverse correlation between the drug concentration and Ser2 phosphorylation, with varying effects observed between KO and wt ovarian cancer cells (Figure 4c, left panel). Once again, we observed that the OVCAR-8 KO cells displayed significantly reduced sensitivity to the CDK9 inhibitor BAY compared to their wt counterparts (IC_50_ wt vs. KO: 63.87 vs. 107.10 nM) (Figure 4c, right panel, Supplementary Figure S3c).

Given that BRD4 was shown to be upregulated in HGSOC cancer cell lines, it is important to note that, in recent years, BRD4 has emerged as a potential target for cancer therapy. Several small molecule inhibitors have been developed to target BRD4. These inhibitors are primarily used to disrupt the interaction between BRD4 and acetylated histones, which can lead to the downregulation of specific genes associated with cancer. Based on our observation of the increased BRD4 expression in OVCAR-8 KO cells (Figure 3a,b), we determined the sensitivity of these cells to two small-molecule BRD4 inhibitors—BI894999 (BI) (Figure 4d, Supplementary Figure S3d) and ABBV744 (ABBV) (Figure 4e, Supplementary Figure S3e). These inhibitors disrupt the interaction between BRD4 and actylated histones and have been used in pre-clinical and clinical trials against various cancer entities [24,25,26,27,28,29]. Similar to our observation with BAY, the OVCAR-8 KO cells were significantly less sensitive to both BI and ABBV than their wt counterpart (IC_50_ wt vs. KO: 0.35 vs. 0.92 µM for BI and 1.22 vs. 2.38 µM for ABBV) (Figure 4d,e). To summarize, these results established that the loss of Caspase-8 expression conferred a resistance to the HGSOC cell line OVCAR-8, not only against standard chemotherapeutics such as Carboplatin and Paclitaxel but also against small-molecule inhibitors targeting CDK9 and BRD4.

### 3.5. Combinations of Chemotherapeutics with BRD4 Inhibitors’ Synergistically Sensitized HGSOC Cells Lacking Caspase-8 Expression

**Figure 5:**
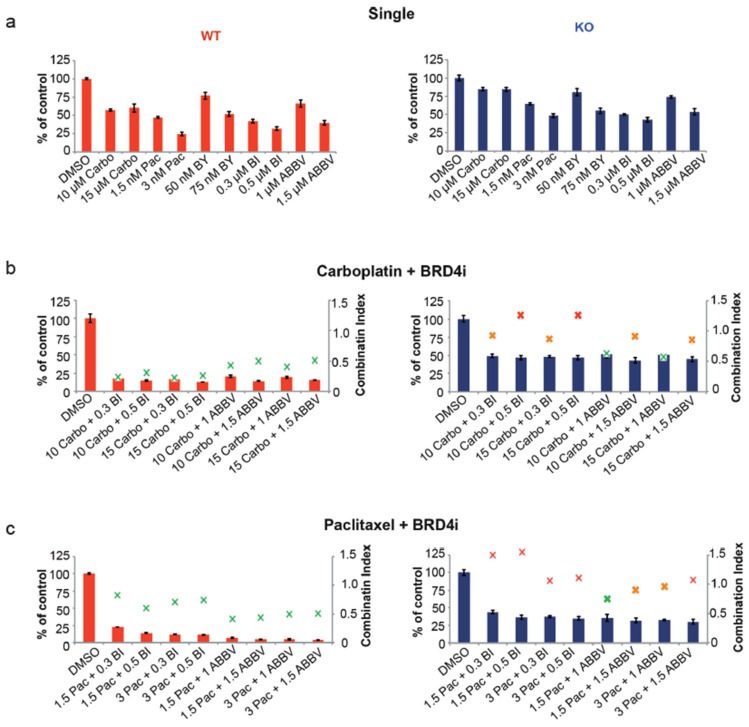

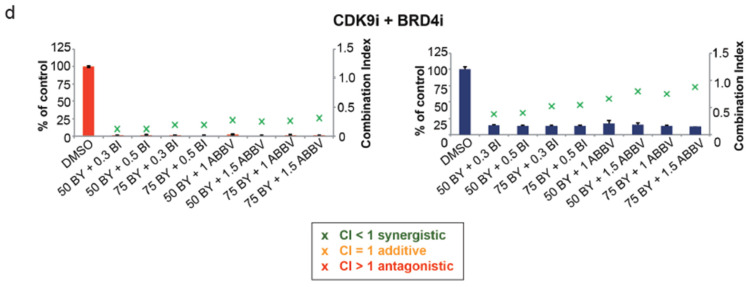
Effects of combination therapy with small-molecule inhibitors of BRD4. (**a**) OVCAR-8 wt and KO cells were treated with two different concentrations of Carboplatin (Carbo µM), Paclitaxel (Pac nM), BAY1251152 (BAY nM), BI894999 (BI µM), and ABBV744 (ABBV µM), and the 2D cell-proliferation was measured using a CellTiter-Blue Cell Viability 96 h post-treatment. (**b**) OVCAR-8 wt and KO cells were treated with a combination of two different concentrations of Carboplatin (µM) and BI894999 (BI µM), or ABBV744 (ABBV µM), and the 2D cell-proliferation was measured using a CellTiter-Blue Cell Viability 96 h post-treatment. (**c**) OVCAR-8 wt and KO cells were treated with a combination of two different concentrations of Paclitaxel (nM) and BI894999 (BI µM), or ABBV744 (ABBV µM), and the 2D cell proliferation was measured using a CellTiter-Blue Cell Viability 96 h post-treatment. (**d**) OVCAR-8 wt and KO cells were treated with a combination of two different concentrations of BAY (nM) and BI894999 (BI µM), or ABBV744 (ABBV µM), and the 2D cell-proliferation was measured using a CellTiter-Blue Cell Viability 96 h post-treatment. The combination Index (CI), representing the integrated effects of these doses on cell survival, was calculated using the CompuSyn software v.1. [*n* = 3]. Displayed are the results of one representative experiment as mean ± SD; CI values > 1 = antagonistic; =1 = additive; and <1 = synergistic].

Our next objective was to determine whether *CASP8^−/−^* KO cells could be sensitized to Carboplatin and Paclitaxel by treating these cells with a combination of these chemotherapeutics with the small-molecule BRD4 inhibitors (BI, ABBV). At first, we treated OVCAR-8 wt and *KO* cells with two different concentrations of Carboplatin, Paclitaxel, BAY, BI, and ABBV, and determined their proliferation over 96 h. KO cells were less sensitive to each treatment than their wt counterparts (Figure 5a, Supplementary Figures S4a and S5a). Additionally, except for Carboplatin, both wt and KO cells showed a concentration-dependent sensitivity toward Paclitaxel, BAY, BI, and ABBV (Figure 5a, Supplementary Figures S4a and S5a).

Next, we treated OVCAR-8 wt and KO cells with combinations of different concentrations of Carboplatin with BI or ABBV. Compared to the single treatment, the effects of the combinatorial treatment were significantly more pronounced, both in wt and KO cells, albeit the inhibition of wt cell proliferation was more robust compared to KO cells. Notably, the Combination Index (CI) calculated, based on the cell proliferation of these cells, demonstrated that the combination of 10 or 15 µM of Carboplatin with 1 µM of ABBV led to the synergistic inhibition of KO cell proliferation (CI = 0.63 and 0.57, respectively) (Figure 5b, Supplementary Figures S4b and S5b). Next, we similarly treated OVCAR-8 wt and KO cells with combinations of different concentrations of Paclitaxel with BI or ABBV. Once again, in comparison to individual treatments, the reduction in cellular proliferation was more pronounced with the combinatorial approach in both wt and KO cells, although wt cells exhibited greater sensitivity than KO cells. The CI values indicated that the best combination was 1.5 nM of Paclitaxel with 1 µM of ABBV, which led to the synergistic inhibition of KO cell proliferation (CI = 0.75) (Figure 5c, Supplementary Figures S4c and 5c).

In our final set of experiments, we explored the impact of combining varying concentrations of the CDK9 inhibitor BAY with the BRD4 inhibitors BI or ABBV in the context of OVCAR-8 wt and KO cells. Notably, these combinations exhibited the most potent inhibition of cell proliferation, with a synergistic effect observed in each combination of BAY with BI and BAY with ABBV (Figure 5d, Supplementary Figures S4d and S5d). In summary, these outcomes underscore the potential to sensitize the more resistant OVCAR-8 ^−/−^ cells to Carboplatin or Paclitaxel treatment through a synergistic approach, effectively overcoming their resistance. Furthermore, the combined use of inhibitors targeting transcriptional elongation, such as small-molecule BRD4 inhibitors alongside a CDK9 inhibitor, presents a highly promising strategy for effectively targeting OVCAR-8 KO cells.

### 3.6. Simultaneous Inhibition of CDK9 and BRD4 Induces Increased Cell Death in Caspase-8 KO Cells

**Figure 6:**
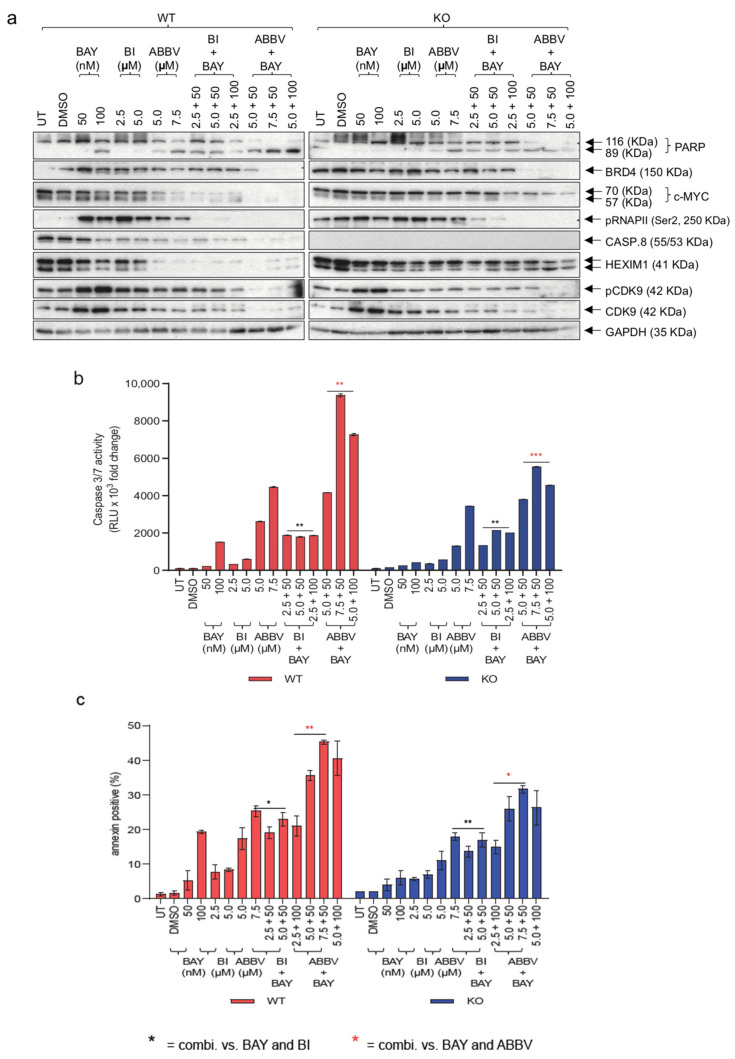
Mechanism of cell death induced by CDK9 and BRD4 small-molecule inhibitors. OVCAR-8 wt and KO cells were treated with two different concentrations of BAY and BI, or ABBV, either alone or with combinations of BAY with BI or BAY with ABBV. (**a**) Immunoblot of the lysates of these cells was checked for the levels of cleaved PARP, BRD4, c-Myc, RNAPII/pSer2 (pCTD), Caspase-8, HEXIM1, pCDK9, CDK9, and GAPDH; (**b**) The measurement of Caspase-3/7 activities using a Caspase-Glo 3/7 assay normalized to their respective DMSO controls. [*n* = 3]. Displayed are the results of one representative experiment indicated as mean ± SD, ** p ≤ 0.01; *** p ≤ 0.001. (**c**) Graphical representation of Annexin V positive (%) cells normalized to their respective DMSO controls. The results are displayed as mean ± SD; *n* = 3 for each concentration; *p*-value (two-way Anova); * *p* ≤ 0.05; ** p ≤ 0.01; *** p ≤ 0.001; * = combi. vs. BAY and BI; Red * = combi. vs. BAY and ABBV]. The original western blots of Figure 6a are shown in Figure S7.

Having demonstrated the effectiveness of sensitizing HGSOC cells lacking Caspase-8 expression using combinations of Carboplatin and Paclitaxel with BRD4 inhibitors, our next objective was to evaluate the mechanisms through which these small-molecule inhibitors induce cell death in OVCAR-8 wt and KO cells. For this purpose, we first treated OVCAR-8 wt and KO cells with two concentrations each of BAY, BI, and ABBV, either alone or in combination (BAY + BI or BAY + ABBV) for 48 h. Immunoblotting of the resulting lysates revealed that, at the concentrations used, ABBV was much more effective at inducing PARP cleavage than BI in wt and KO cells. Additionally, we observed more PARP cleavage in the KO and wt cells, respectively, when treated with combinations of BAY and ABBV, compared to when the KO and wt cells were treated with these inhibitors alone (Figure 6a). This tendency was further substantiated when we assessed Caspase-3/7 activities (Figure 6b), and overall apoptosis in these cells (Figure 6c).

Of note, we conducted an analysis of HEXIM1 expression, a gene that has consistently exhibited a robust and consistent modulation in response to Bromodomain and Extra terminal domain (BET) inhibitors across diverse cancer indications [30]. We observed that HEXIM1 was downregulated much more in the wt cells than the KO cells, emphasizing the resistance of the KO cells to BRD4 inhibition. Also, ABBV was much more efficient at this downregulation than BI, although when combined with BAY, both BI and ABBV proved equally efficient at downregulating HEXIM1 (Figure 6a). This trend was similarly reflected in the phosphorylation of the Ser2 residue of RNAPII CTD, indicative of inhibition in transcription elongation, and this effect was also mirrored in the expression of c-Myc, a transcription factor integral to the RNAPII-mediated transcription elongation [31] (Figure 6a).

The disruption of a specific gene, such as Caspase-8 induced by CRISPR/Cas9, can potentially activate compensatory mechanisms, thereby complicating the accurate interpretation of results. Thus, we performed a transient knockdown by RNAi and investigated the impact of different inhibitors on wt and KO cells. We first knocked down *CASP8* expression in wt cells with siRNA (siCasp8). Control siRNA (siCtrl) transfected cells were used as a negative control. Next, we treated both cell types with BAY and ABBV, either alone or in combination (Supplementary Figure S6a). Not only were the siCasp8 transfected cells more resistant to the combination of BAY and ABBV, showing less PARP cleavage (Supplementary Figure S6a), Caspase-3/7 activities (Supplementary Figure S6b) and less overall apoptosis (Supplementary Figure S6c). Taken together, these findings in the KO cells corroborated and reinforced the previous data obtained with KO cells.

In conclusion, these findings substantiate the proposed model wherein the absence of Caspase-8 expression conveys a resistance to KO cells by increasing the phosphorylation of CDK9 at Thr187 and elevating the BRD4 expression through non-apoptotic mechanisms. Notably, this resistance can be effectively overcome by employing combinations of inhibitors targeting CDK9 and BRD4. Moreover, Caspase-8 is pivotal in the cell death induced by BRD4 inhibitors but not CDK9 inhibitors.

## 4. Discussion

The doublet of Carboplatin and Paclitaxel is still considered the chemotherapy of choice for treating ovarian cancer, with 95% of women diagnosed with ovarian cancer receiving this regimen [32]. Various trials have established the combination of Carboplatin and Paclitaxel to be well tolerated by advanced ovarian cancer patients, with a median PFS of 13.6–19.3 months [33]. This combination has a more favorable toxicity profile, better quality of life, and requires a shorter schedule than the combination of Cisplatin with Paclitaxel [33,34,35]. However, tumor recurrence following the first-line chemotherapy with Carboplatin and Paclitaxel is detected in 25% of early-stage and 80% of advanced ovarian cancer patients, with disease relapse occurring within 2 years of initial treatment in a majority of advanced ovarian cancer patients [33,36]. Platinum-resistant ovarian cancer is one of the biggest challenges in treating patients who relapse within 6 months of completing first-line chemotherapy, indicating a low response rate to subsequent chemotherapy, PFS of 3–4 months, and median survival of >1 year [37]. Paclitaxel resistance has been reported in >70% of patients at the time of diagnosis and in ~100% of relapsed patients [38]. Hence, identifying novel biomarkers and resistance mechanisms is critical to overcoming the resistance, developing newer treatment strategies, and improving patient survival.

Our work here has demonstrated that the low expression of Caspase-8 in ovarian cancer patients has a significantly poorer prognosis compared to Caspase-8-expressing patients. Although both the canonical and non-canonical roles of caspase 8 are likely to influence patient survival, the precise impact of each function remains to be determined. We have also demonstrated that HGSOC OVCAR-8 cells lacking a Caspase-8 expression are more resistant to Carboplatin and Paclitaxel under 2D growth conditions. Interestingly, we observed an increase in the level of BRD4 and an elevated level of RNAPII phosphorylation of Ser-2 in ovarian cancer KO cells lines. Moreover, in both OVCAR-3 and OVCAR-8 cell lines, we observed the increase in the level of BRD4 in the nucleus of the KO cells compared to their wt counterparts. BRD4 plays a very critical role in the RNAPII-mediated transcription. At the onset of the productive transcription elongation, BRD4 recruits the active CDK9/Cyclin T1 complex (P-TEFb) from its negative regulatory complex 7SK snRNP to the RNAPII, where it can phosphorylate the C-Terminal Domain (CTD) of RNAPII at Ser2. Additionally, the kinase activity of the BRD4-bound P-TEFb is kept under check due to the phosphorylation of CDK9 at Thr29. The recruitment of the PP2α phosphatase results in the de-phosphorylation of Thr29 to finally ‘release’ the total kinase activity of CDK9 [13,39]. Our observations provide evidence that the downregulation of Caspase-8 or its knockdown leads to an activation of RNAPII promoting the transcriptional elongation of HGSOC cancer cells. The overexpression of BRD4, at both genomic and protein levels, has been reported in HGSOC cells, resulting in a more aggressive tumor phenotype and poor prognosis [40]. Most importantly, we could show that combining BRD4 small-molecule inhibitors synergistically enhanced the 2D cell proliferation inhibitory effect of both Carboplatin and Paclitaxel. This finding is encouraging, as a phase Ib/IIa clinical trial of a combination of Carboplatin and the oral BET inhibitor PLX2853 to treat platinum-resistant ovarian cancers has recently been reported [41]. Other reports have also confirmed the synergistic effect of BET inhibitors on Cisplatin, Carboplatin, and Paclitaxel against ovarian cancer [42], NSCLC [43], prostate cancer [44], and breast cancer [45].

Beyond non-apoptotic functions of Caspase-8, Caspase-8 is a crucial initiator of apoptosis, the programmed cell death process [46,47]. It acts as a pivotal point in extrinsic apoptosis, triggered by external signals like Fas ligand binding. Once activated, Caspase-8 cleaves downstream effector caspases, leading to the activation of a cascade of events that result in cell dismantling. Moreover, Caspase-8’s involvement extends beyond apoptosis; it plays a role in other cell death pathways, including necroptosis and pyroptosis, contributing to cell fate decisions. Caspase-8 is expressed as zymogen, which represents an inactive precursor. In our studies using different ovarian cancer cell lines treated with various chemotherapeutics, signs of the maturation cleavage of Caspase-8 could not be detected, suggesting that the classical role of Caspase-8 as an initiator of the extrinsic pathway plays a minor role for the overall cell death measured in our experiments.

Hormonal therapy emerges as a beneficial option for managing patients with estrogen-receptor (ER) positive advanced ovarian cancer [48], a patient subset represented in our study by the ER-expressing cell line OVCAR-3. Findings indicate that estrogen receptors may play a role in apoptotic pathways involving Caspase-8, and disruptions in these pathways might influence the response to hormonal therapies and chemotherapy. Furthermore, estrogen receptors have demonstrated interactions with the Fas ligand promoter in human monocytes, and the estrogen impact on monocyte cell survival involves the regulation of Fas or FasL expression [49]. This implies that estrogen receptors may influence apoptotic pathways involving Caspase-8, a crucial mediator of apoptosis.

Moreover, a study has demonstrated that estrogen-related hormones can induce apoptosis by stabilizing Schlafen-12 protein turnover, and this process engages Caspase-8 and Caspase-9 [50]. This underscores the direct involvement of Caspase-8 in the apoptotic pathways induced by estrogen-related hormones. Consequently, these data propose that the Caspase-8 expression could serve as a valuable factor for patient stratification and predicting the effectiveness of the recommended hormonal therapy for ovarian cancer patients.

Our current work has demonstrated that the small-molecule CDK9 inhibitor BAY1251152 as well as the BRD4 inhibitors BI894999 and ABBV744, induce cell death in HGSOC cancer cells. Therefore, a combination of BAY1251152 with either BI894999 or ABBV744 (especially with ABBV744) could significantly and synergistically overcome the effects of the BRD4 inhibitors in OVCAR-8 cells without Caspase-8 expression. Our data proves the veracity of combining BRD4 and CDK9 small-molecule inhibitors to target the HGSOC cell lines effectively. This treatment strategy has been or is being tested against several cancer entities [13,51,52,53].

Summing up our work, we are proposing three strategies for an in depth investigation in preclinical models of ovarian cancer using immune-compromised mice and cells that exhibit low Caspase-8 expression and resistance to Carboplatin and/or Paclitaxel—combinations of (1) Carboplatin with small-molecule BRD4 inhibitors; (2) Paclitaxel with small-molecule BRD4 inhibitors; and (3) small-molecule BRD4 and CDK9 inhibitors. In addition, we are also proposing two predictive markers of chemoresistance—BRD4 and pCDK9.

## 5. Conclusions

Our study sheds light on the challenges associated with treating high-grade serous ovarian cancer (HGSOC), particularly regarding the Platinum/Taxane resistance commonly observed in patients. Identifying the low Caspase-8 expression as a biomarker associated with a poor prognosis and resistance to chemotherapy provides valuable insights into the underlying mechanisms involved in HGSOC. The study reveals that the Caspase-8 expression influences the transcriptional regulation of HGSOC cells, leading to the increased BRD4 expression and CDK9 activity.

The proposed therapeutic strategies, including the combination of Carboplatin with small-molecule BRD4 inhibitors, Paclitaxel with small-molecule BRD4 inhibitors, and the use of small-molecule BRD4 and CDK9 inhibitors, offer promising prospects for sensitizing cells with reduced Caspase-8 to undergo cell death. These synergistic approaches have the potential to overcome chemoresistance and improve the outcomes for advanced ovarian cancer patients.

Finally, the study proposes BRD4 and pCDK9 as predictive markers of chemoresistance, providing clinicians with valuable tools for identifying patients who may benefit from alternative treatment strategies.

Overall, the current study contributes to our understanding of HGSOC and offers potential therapeutic interventions that could significantly impact the management of this lethal gynecological cancer.

## Figures and Tables

**Table 1:** Low CASP8 expression was associated with higher clinical stages but not tumor grade.

		CASP8 Expression			
		Low	High	Total	*p*
Age > 59 year	N	64	71	135	0.37
	Y	58	50	108	
Grade	1	1	0	1	0.66
	2	12	10	22	
	3	105	108	213	
	4	0	1	1	
Stage	1	1	5	6	0.007
	2	5	17	22	
	3	96	87	183	
	4	20	12	32	
High Stage	N	6	22	28	0.0012
	Y	116	99	215	

## Data Availability

The Data is contained within the article or Supplementary Material.

## Supplementary Materials

The following supporting information can be downloaded at: https://www.mdpi.com/article/10.3390/cancers16010107/s1, Supplementary Figure S1: Schematic representation of transcriptional elongation by RNA polymerase II and its regulation. Supplementary Figure S2: Knock-out of Caspase-8 expression in OVCAR-3 cells enhances CDK9 phosphorylation and BRD4 expression. Supplementary Figure S3: Effects of Caspase-8 knock-out on the response of ovarian cancer cells to Carboplatin, Paclitaxel, and small-molecule inhibitors of CDK9 and BRD4. Supplementary Figure S4: Effects of combination therapy with small-molecule inhibitors of BRD4. Supplementary Figure S5: Effects of combination therapy with small-molecule inhibitors of BRD4. Supplementary Figure S6: The transient downregulation of Casp8 using siRNA confers resistance to BRD4 and CDK9 inhibitors in OVCAR-8 cells. Supplementary Figure S7: Original Western Blot images.
