# Supplementary material for: Synergistic Sensitization of High-Grade Serous Ovarian Cancer Cells Lacking Caspase-8 Expression to Chemotherapeutics Using Combinations of Small-Molecule BRD4 and CDK9 Inhibitors
Source: Cancers (Basel). 2023 Dec 24;16(1):107. doi: 10.3390/cancers16010107 (PMC10778249; doi:10.3390/cancers16010107)
Supplement: Supplementary file 1 [file cancers-16-00107-s001.zip › cancers-2743728-Figure S7.pdf]

Unropped Figures

a

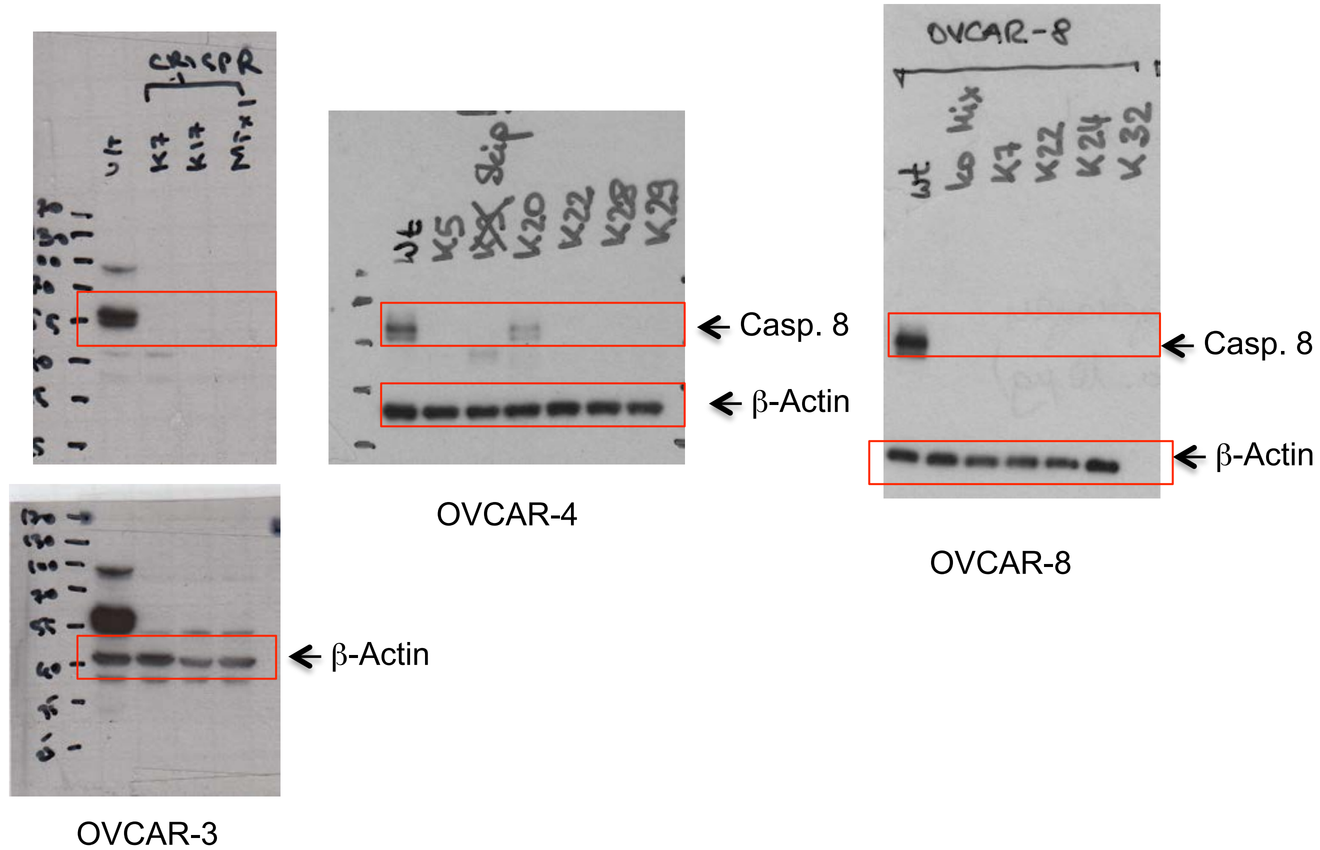

Figure S2

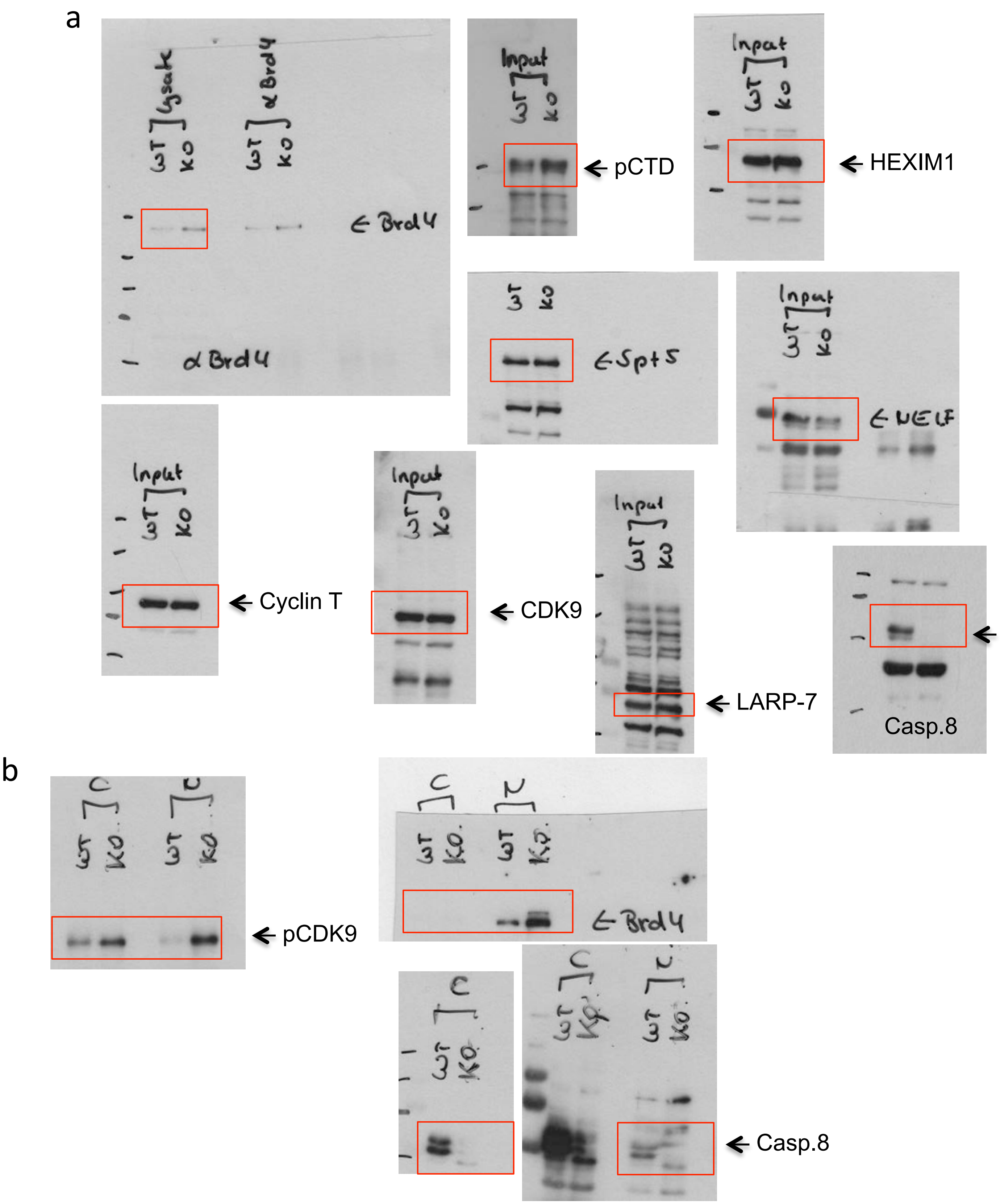

Figure S3

Figure S3

c

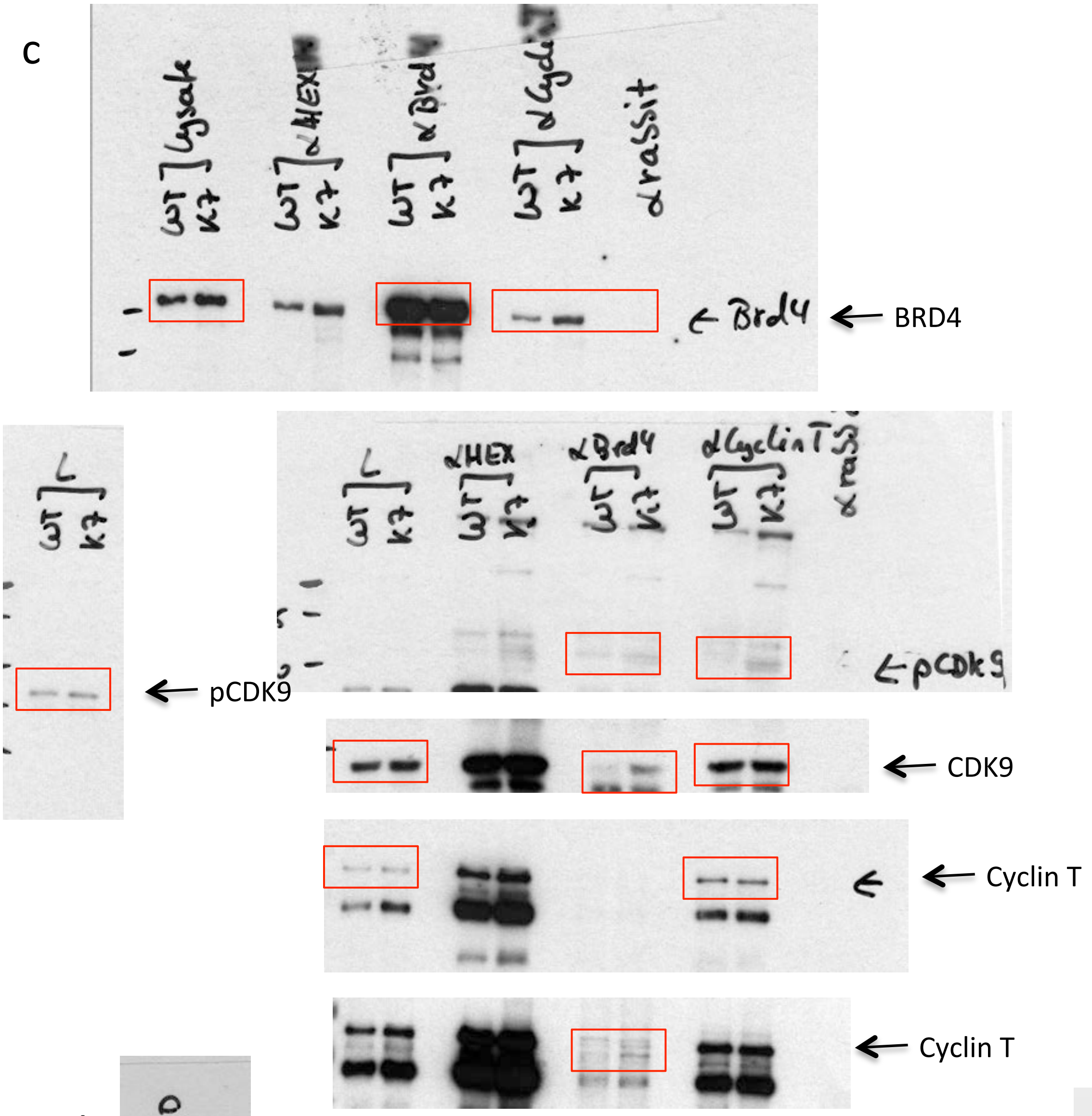

d

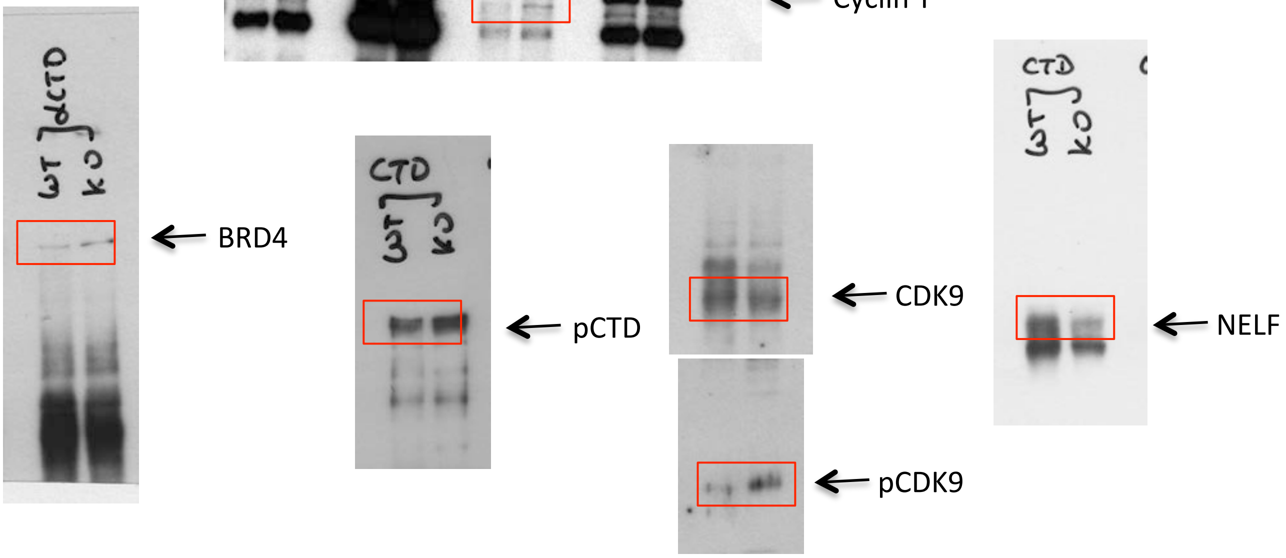

c

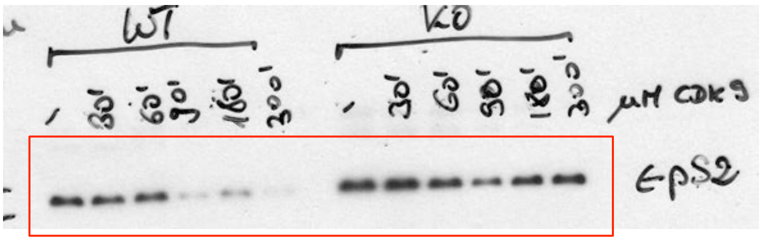

Figure S4

Figure S6

a

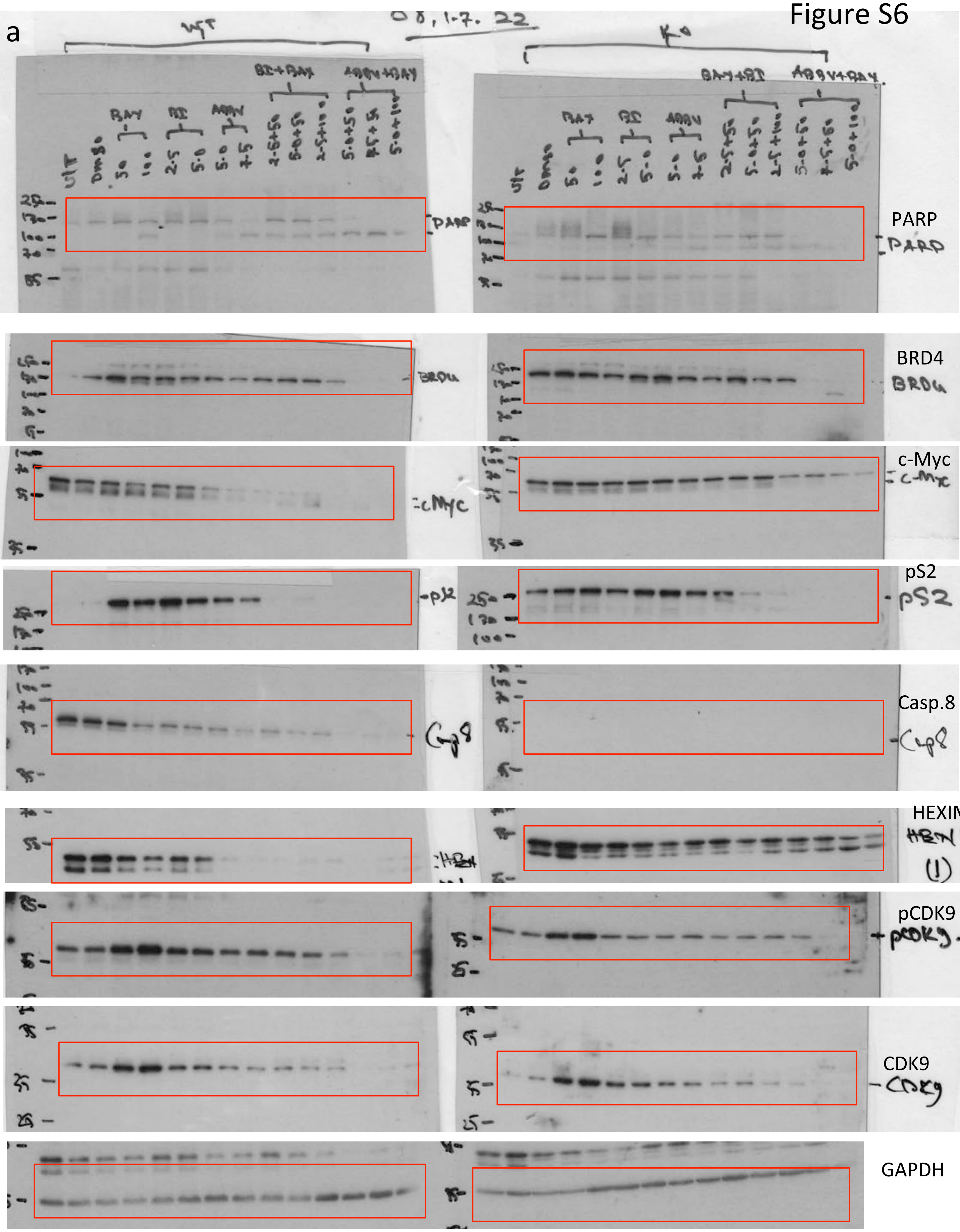

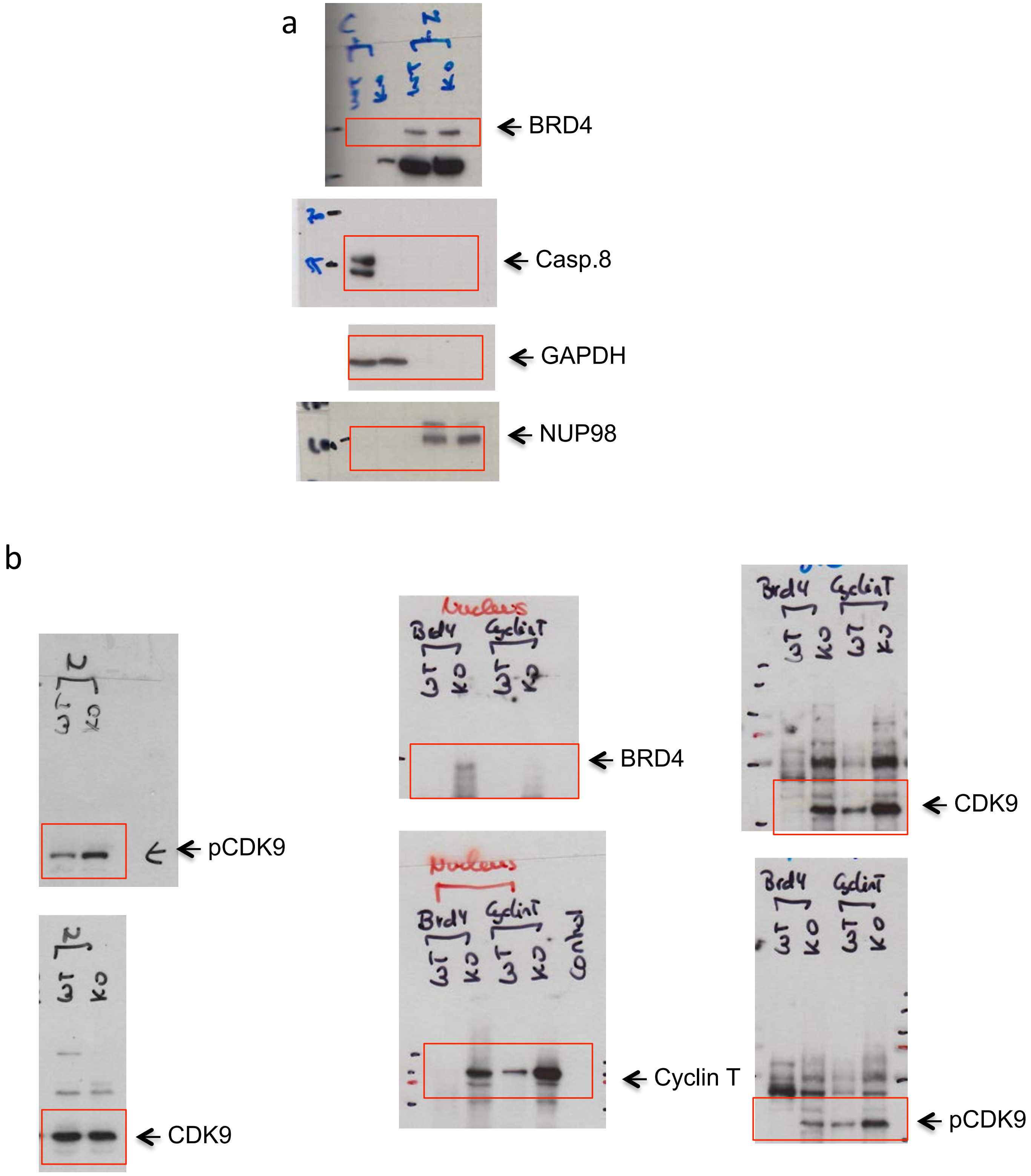

Supplementary Figure S2

a

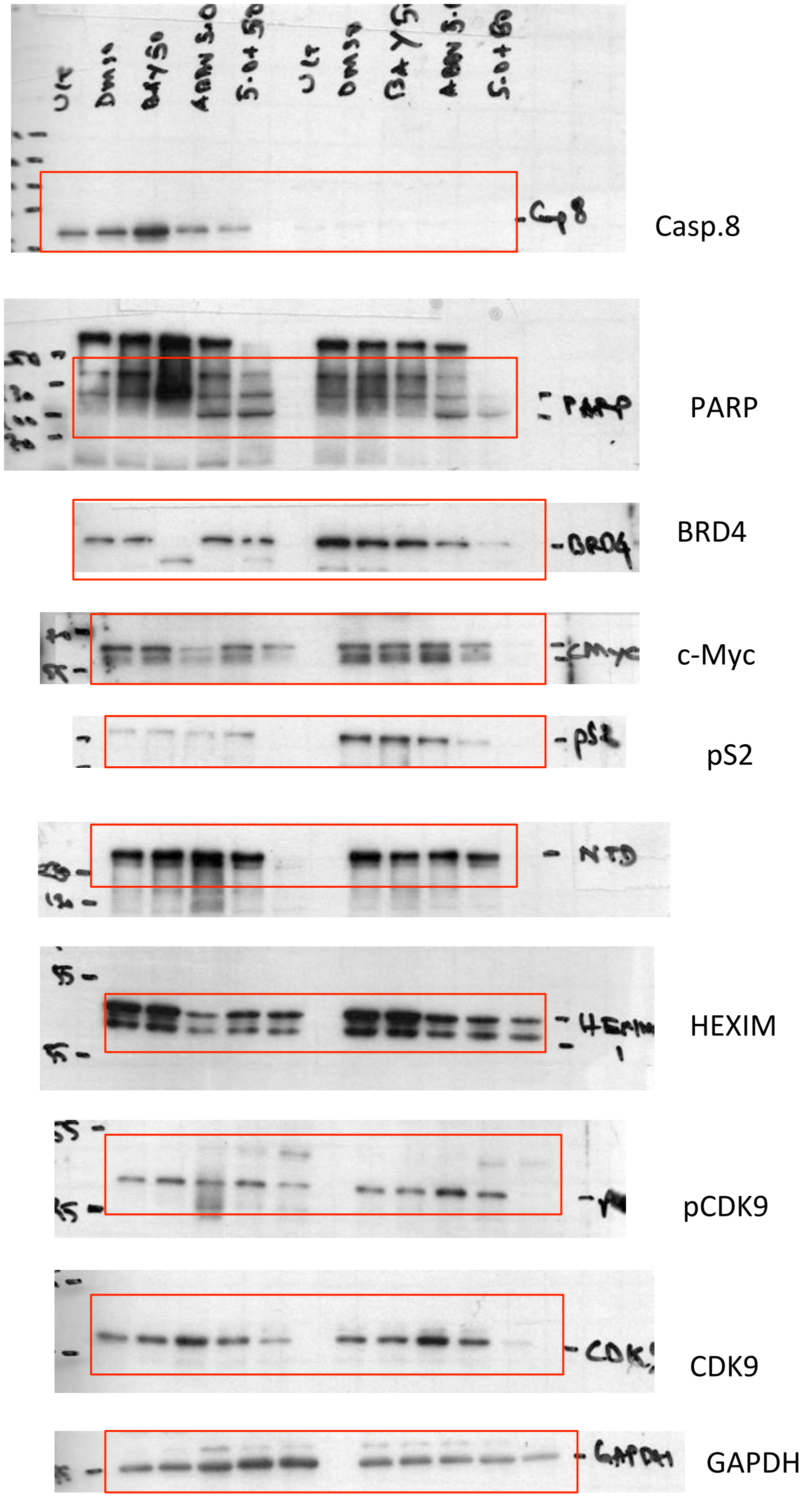

Supplementary Figure S3
