# Supplementary material for: Synergistic Sensitization of High-Grade Serous Ovarian Cancer Cells Lacking Caspase-8 Expression to Chemotherapeutics Using Combinations of Small-Molecule BRD4 and CDK9 Inhibitors
Source: Cancers (Basel). 2023 Dec 24;16(1):107. doi: 10.3390/cancers16010107 (PMC10778249; doi:10.3390/cancers16010107)
Supplement: Supplementary file 1 [file cancers-16-00107-s001.zip › cancers-2743728-supplementary.pdf]

a

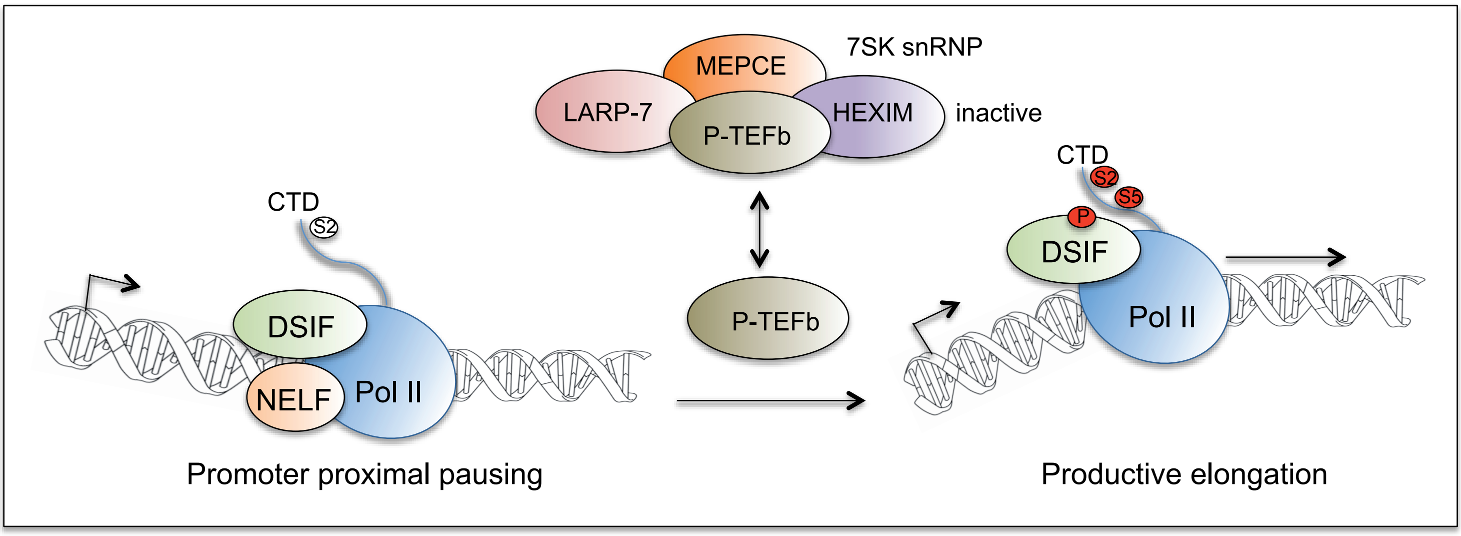

b

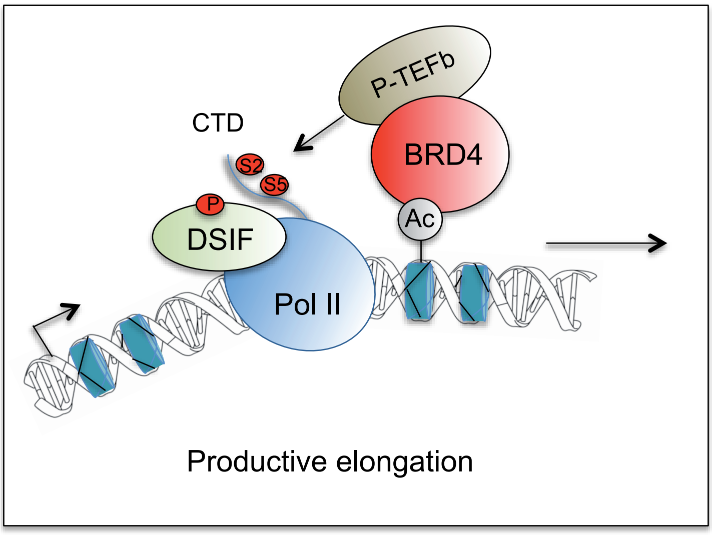

Supplementary Figure S1

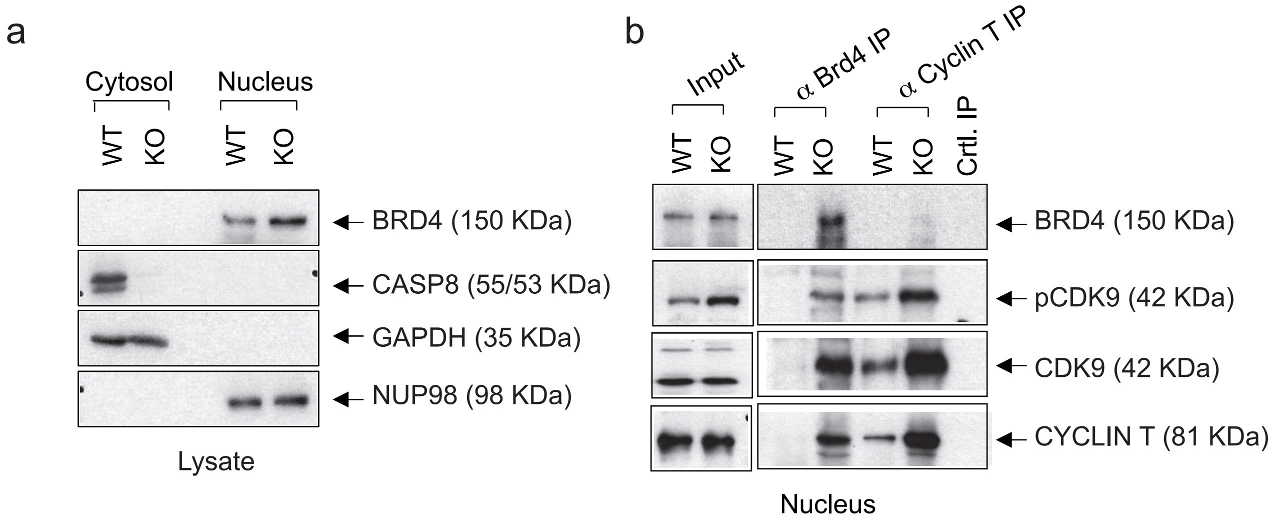

Supplementary Figure S2

a

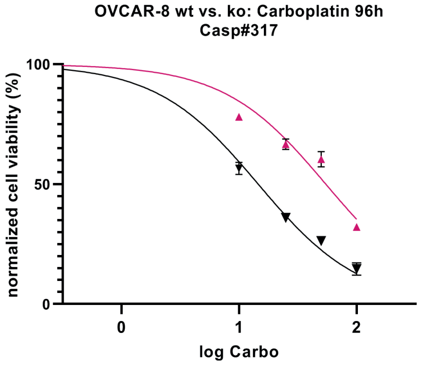

OVCAR-8 wt: LogIC50 1.163 IC50 14.56 µM
OVCAR-8 ko: LogIC50 1.738 IC50 54.75 µM

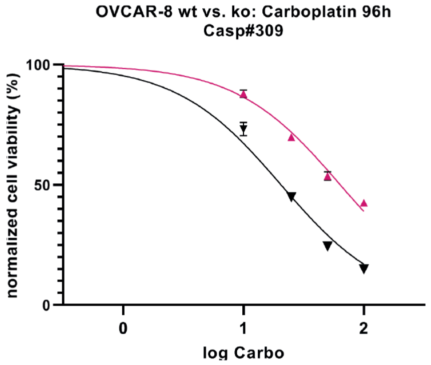

IC50: OVCAR-8 wt 20.5 µM
OVCAR-8 ko 63.8 µM

b

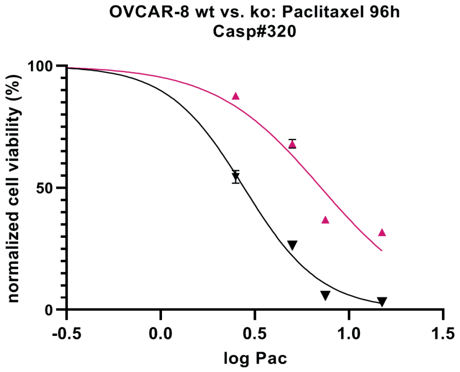

OVCAR-8 wt: IC50 2.774 nM
OVCAR-8 ko: IC50 7.119 nM

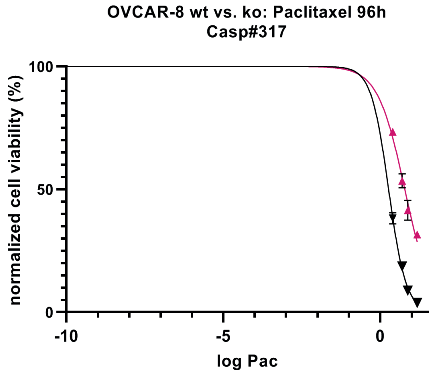

OVCAR-8 wt: IC50 1.849 nM
OVCAR-8 ko: IC50 6.059 nM

c

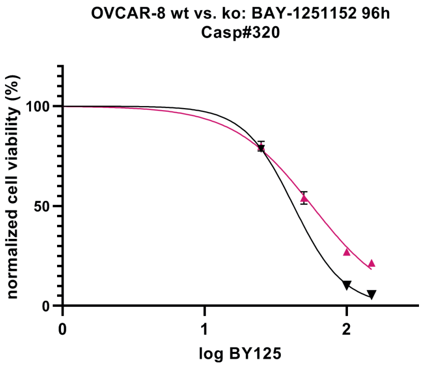

OVCAR-8 wt: IC50 42.4 nM
OVCAR-8 ko: IC50 57.1 nM

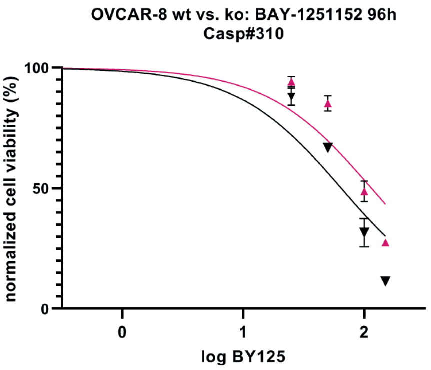

IC50: OVCAR-8 wt 64.6 nM
OVCAR-8 ko 114.8 nM

d

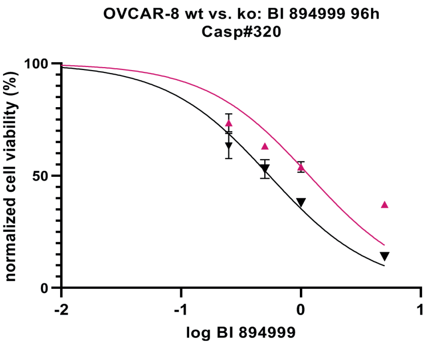

OVCAR-8 wt IC50 0.546 µM
OVCAR-8 ko IC50 1.176 µM

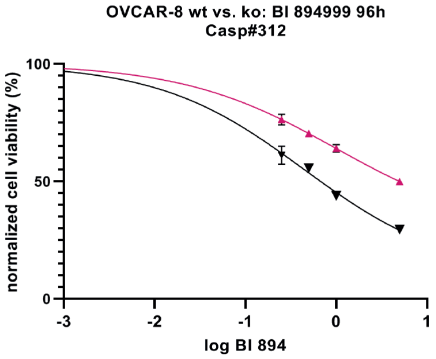

IC50: OVCAR-8 wt 0.442 µM
OVCAR-8 ko 1.001 µM

e

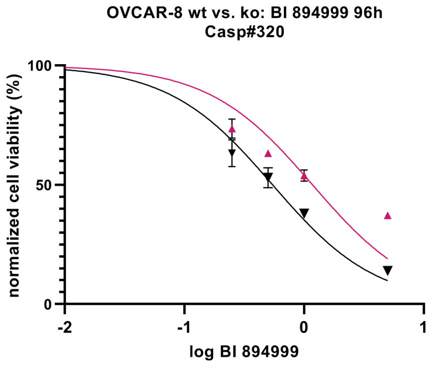

OVCAR-8 wt IC50 0.546 µM
OVCAR-8 ko IC50 1.176 µM

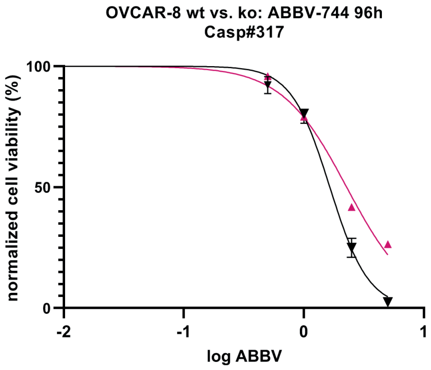

OVCAR-8 wt: IC50 1.65 µM
OVCAR-8 ko: IC50 2.27 µM

Supplementary Figure S3

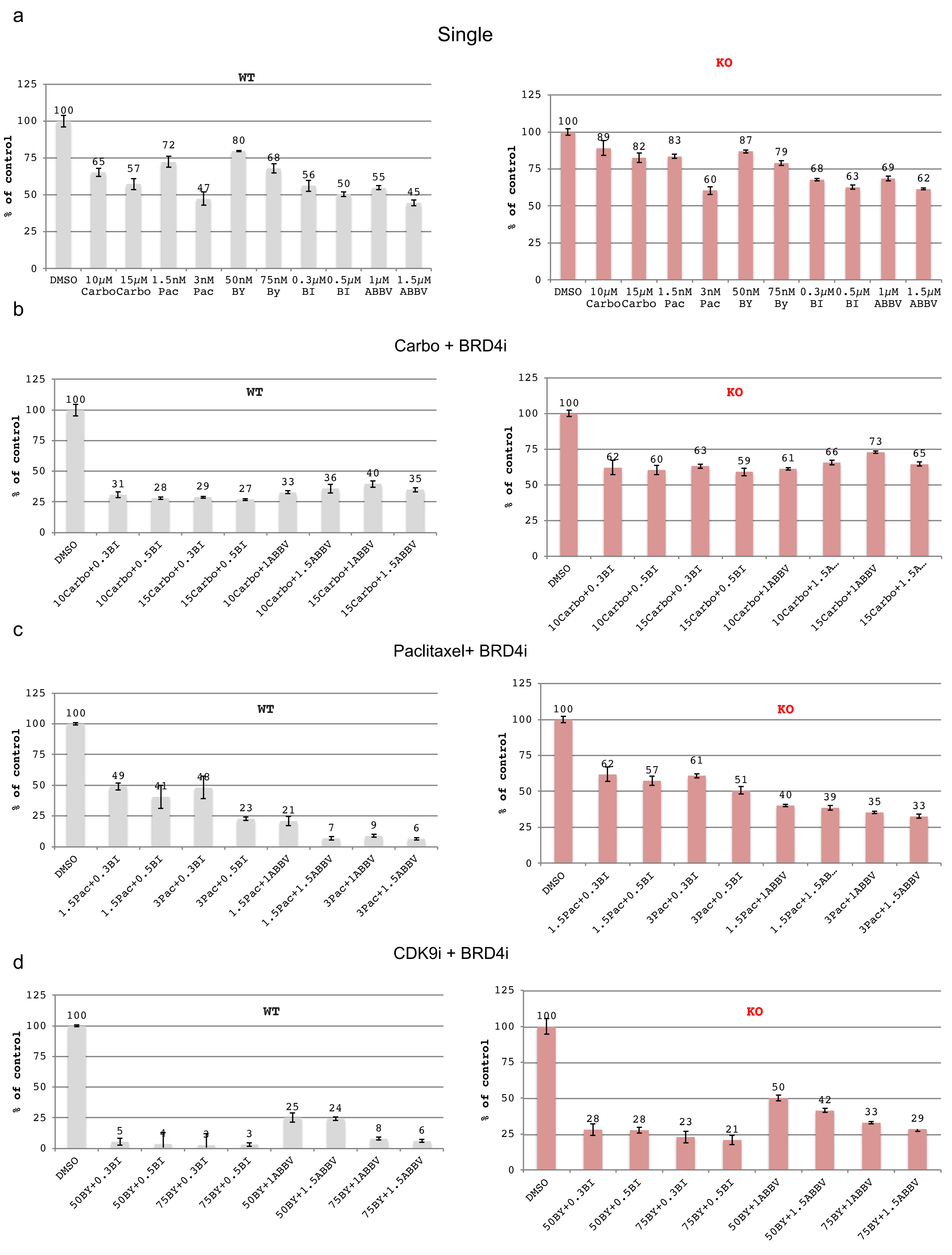

Supplementary Figure S4

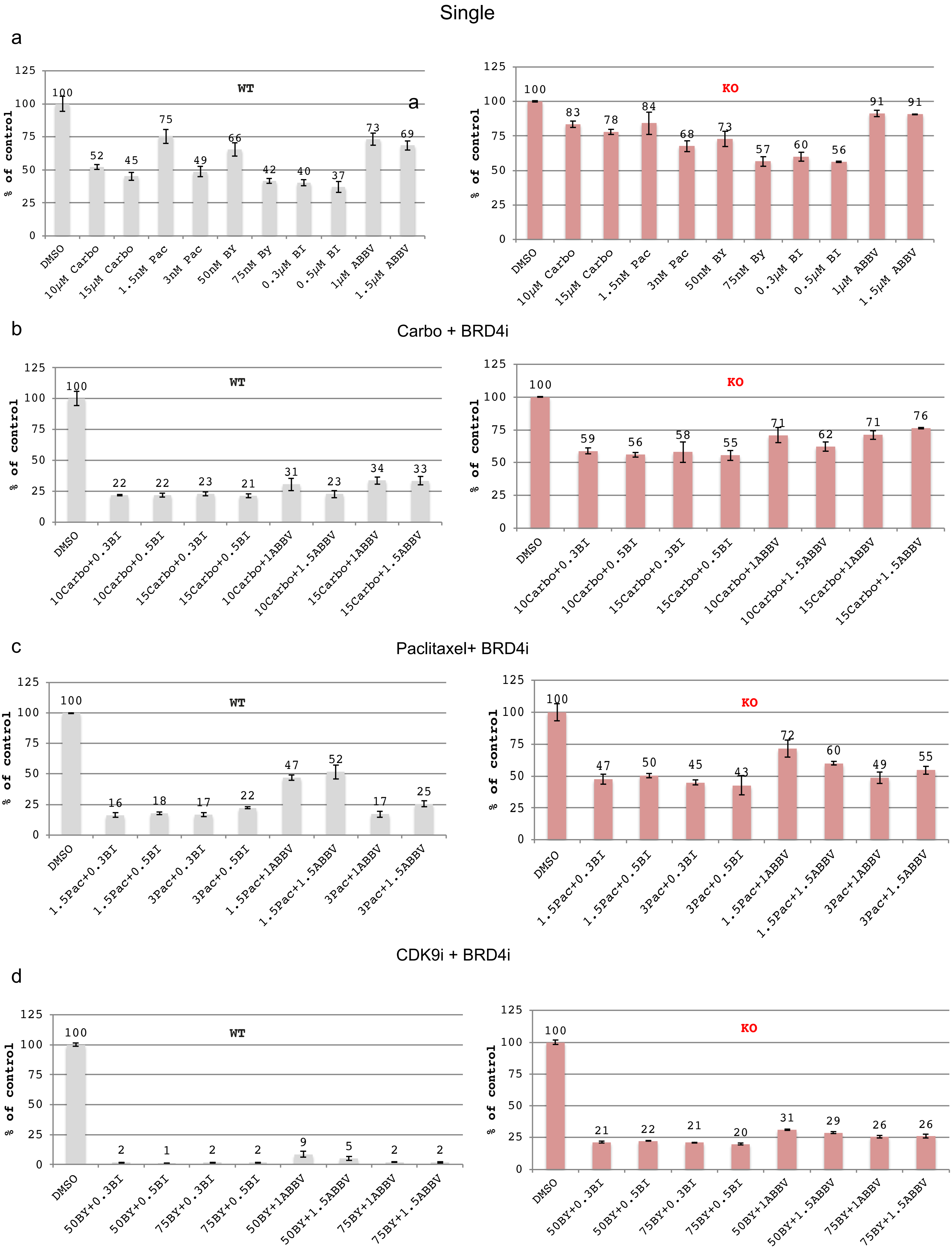

Supplementary Figure S5

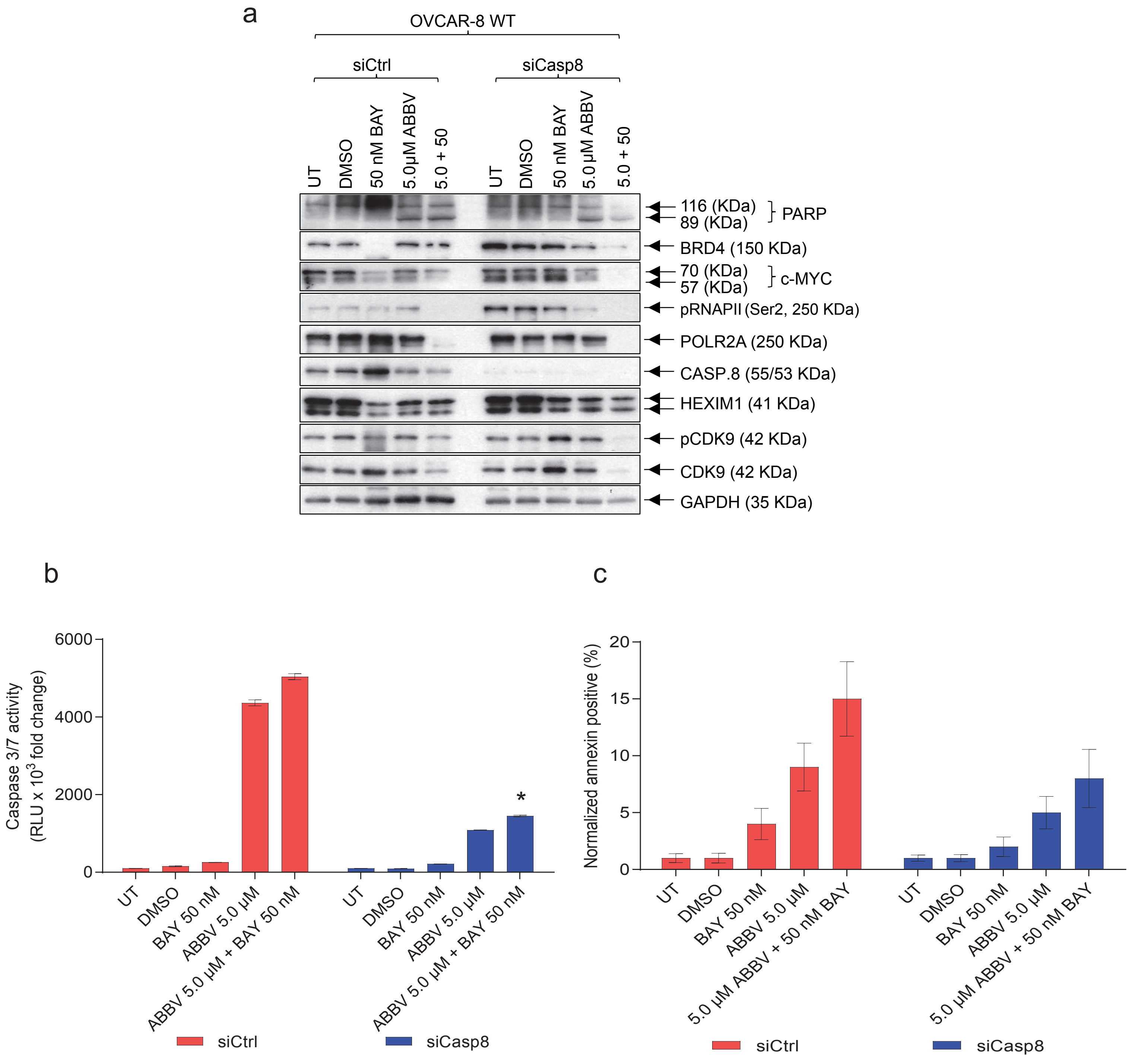

* = combi. siCtrl vs. combi. siCasp8

Supplementary Figure S6
